# Selection and Use of Manganese Dioxide by Neanderthals

**DOI:** 10.1038/srep22159

**Published:** 2016-02-29

**Authors:** Peter J. Heyes, Konstantinos Anastasakis, Wiebren de Jong, Annelies van Hoesel, Wil Roebroeks, Marie Soressi

**Affiliations:** 1 Faculty of Archaeology, Leiden University, Einsteinweg 2, 2333CC Leiden The Netherlands; 2 3mE Process and Energy, Delft University of Technology, Leeghwaterstraat 39, 2628CB, Delft, The Netherlands

## Abstract

Several Mousterian sites in France have yielded large numbers of small black *blocs*. The usual interpretation is that these ‘manganese oxides’ were collected for their colouring properties and used in body decoration, potentially for symbolic expression. Neanderthals habitually used fire and if they needed black material for decoration, soot and charcoal were readily available, whereas obtaining manganese oxides would have incurred considerably higher costs. Compositional analyses lead us to infer that late Neanderthals at Pech-de-l’Azé I were deliberately selecting manganese dioxide. Combustion experiments and thermo-gravimetric measurements demonstrate that manganese dioxide reduces wood’s auto-ignition temperature and substantially increases the rate of char combustion, leading us to conclude that the most beneficial use for manganese dioxide was in fire-making. With archaeological evidence for fire places and the conversion of the manganese dioxide to powder, we argue that Neanderthals at Pech-de-l’Azé I used manganese dioxide in fire-making and produced fire on demand.

Whether the Neanderthal archaeological record testifies to the kind of symbolic behaviours that are considered typical for ‘modern’ humans is a highly debated topic within palaeoanthropology, with the use of coloured materials such as ochres and manganese oxides one of the possible indicators of such behaviours^1^,^2^. More than forty Middle Palaeolithic sites in Europe have yielded coloured mineral materials, the majority dating to the end of the Middle Palaeolithic, between 60,000 and 40,000 years ago^3^. Several Mousterian and Châtelperronian sites in France have yielded large numbers of small black *blocs* (Fig. 1 and Supplementary Information 1) and the usual interpretation is that these are manganese oxides collected for their colouring properties, perhaps for body decoration and potentially for social communication and symbolic expression^3^,^4^,^5^,^6^,^7^. It has been argued that in African Middle Stone Age contexts, ochres were used as a cosmetic pigment in female reproductive strategies^8^ but in extending the hypothesis to the European late Middle Palaeolithic sites, the presence of black “manganese” was interpreted as a Neanderthal female aversion to using the colour red during glacial cycles when, it is argued, pair-bond stability would have been critical^8^. Ethnographic data^9^,^10^,^11^ as well as other evidence^12^,^13^ underline the limitations of exclusive interpretations as pigments and/or their use as symbolic mediators whilst Neanderthal use of ochre more than 200,000 years ago^14^ shows that the manipulation of these materials has a significant time depth in the Middle Palaeolithic. In stark contrast with the wide variety of uses documented for iron oxides, the archaeological and ethnographic records contain very limited evidence for the use of manganese oxides^15^,^16^, all associated with decoration.

Thus far, researchers have focused exclusively on the colour properties of manganese oxides, however, decorative use might imply that any black material soft enough to mark and resilient enough for the mark to remain could have been used. Indeed, both carbon-rich materials and black manganese ores were used in the production of Upper Palaeolithic cave art^17^,^18^,^19^,^20^,^21^,^22^,^23^,^24^. Neanderthals habitually used fire^25^ and if they needed a black material for body decoration, carbon-rich materials such as soot and charcoal were readily available. The use of such carbon-rich materials for body decoration is documented in the ethnographic literature^15^. In contrast to these fire residues, manganese oxides would have had to have been sourced and transported, at considerably higher costs. An explanation of such investments should take into consideration either the possibility of special colouring properties or the chemical properties of manganese oxides, including their oxidising and catalytic properties.

In exploring the potential uses, we have studied the Neanderthal site of Pech-de-l’Azé I where several hundred small black *blocs* (Fig. 1) were uncovered from a Mousterian of Acheulean Tradition layer that was excavated by Bordes in 1954–55 and 1970–71 and more recently by Soressi in 2004–5^26^. The total weight of curated *blocs* from the site is approximately 0.75 kg. Layer 4, sealed by three metres of undisturbed Middle Palaeolithic deposits, with no Upper Palaeolithic deposit present at the site (Supplementary Information 1), was called “*le niveau de foyer”* by Bordes^27^ because of the abundant presence of combustion features, also identified in the recent excavations. The clayey sand matrix of this layer contained stone artefacts, faunal remains, a juvenile Neanderthal tooth and the remains of various individual combustion features^26^,^28^. Single grain optically stimulated luminescence dating gave a weighted mean age of 51.4 + /−2.0 thousand years ago^29^. This age is consistent with conventional radiocarbon, electron-spin resonance and coupled electron-spin resonance/uranium-series ages^30^. Pech-de-l’Azé I was occupied by Neanderthals several millennia before the first evidence for anatomically modern humans in Europe^31^.

Of the large number of *blocs* from Pech-de-l’Azé I available for study^26^, a majority show clear facets of use^6^. The facets display striations related to the abrasion of the *blocs* on a grindstone and part of one sandstone grindstone still covered with black residues was recovered^26^ (Supplementary Information 1, Fig. 1). While the abrasion would have produced powder, some of the *blocs* may have been used to mark soft materials such as animal or human skin^3^. The majority of the Pech-de-l’Azé I manganese ore finds come from Layer 4, but the upper levels also yielded black *blocs*^26^. Pech-de-l’Azé I is not unique and Table 1 in Supplementary Information 1 lists other Mousterian sites in France that are reported to have produced ‘manganese ore’ *blocs* and preserve evidence of combustion features.

Detailed compositions of black materials from Pech-de-l’Azé I and other Middle Palaeolithic sites are not available but it has been inferred that they are manganese oxides. Similar black materials from Châtelperronian contexts have been shown to be predominantly manganese dioxide with a pyrolusite (β-MnO_2_) structure but in one case at Roc-de-Combe, romanèchite (a hydrated barium manganese oxide) was present^7^. Both manganese dioxide and romanèchite are present in the limestone karst close to Pech-de-l’Azé I^32^ and a range of complex manganese oxides have been found in Upper Palaeolithic contexts in the Dordogne region^17^,^19^,^33^,^34^. Evidently a range of different manganese oxides was available to hunter gatherers in that area. The manganese oxide materials from the Châtelperronian of the Grotte-du-Renne at Arcy-sur-Cure, Yonne, approximately 400 kilometres northeast of the Dordogne, were predominantly manganese dioxide as pyrolusite^7^,^15^ and had been ground to powder close to features usually interpreted as fire places.

We sought evidence to assess whether Neanderthals were selecting a specific manganese oxide from a range of manganese ores in the region and if so, whether this material is more effective in other functions than materials that Neanderthals did not select. We report here on a series of compositional analyses, combustion experiments and thermo-gravimetric measurements for three commercial manganese dioxide grades, a barium manganese oxide compositionally similar to materials found in the Dordogne region and three black manganese oxide *blocs* from a Neanderthal context at the Pech-de-l’Azé I site in the Dordogne region of south-western France.

## Results

### Compositions and Structures of the Pech-de-l’Azé I *Blocs*

With the exception of sample PAI-G8-1100, the X-ray fluorescence (XRF) and X-ray Diffraction (XRD) analyses of the twenty four *blocs* from Pech-de-l’Azé I are consistent with a composition of manganese dioxide (MnO_2_). All the *blocs* contain minor amounts of other elements, principally calcium, aluminium, iron, silicon and barium (Supplementary Information 2). Twenty two of the *blocs* have a β-MnO_2_ (pyrolusite) structure, two have no discernible crystal structure and a majority of *blocs* have a small manganite (ϒ-MnOOH) content (Supplementary Information 2). *Bloc* PAI-G8-1100 contains manganese dioxide and has a relatively high barium content but neither it nor the other *blocs* contains romanèchite, a hydrated barium manganese oxide mineral found in the Dordogne region.

The three *blocs* from the excavation spoil at Pech-de-l’Azé I used in the combustion experiments were from the same compositional population as *blocs* from archaeological contexts; their combustion behaviour is probably representative of the behaviour of all the *blocs* except PAI-G8-1100, but this remains to be tested. There is significant *bloc* to *bloc* variability in the compositions and evidence for within-*bloc* variability but the variation is not consistent. The between-*bloc* differences in elements such as arsenic, barium and cobalt that are not likely to be associated with surface contamination, are statistically significant (Supplementary Information 2). There are also differences in manganite content and crystal structure.

The Pech-de-l’Azé I *blocs* differ in minor element contents from the natural ‘manganese oxide’ materials found in the limestone near Pech-de-l’Azé (Supplementary Information 2) and from other ‘complex manganese oxides’ such as the hollandite, todorokite and romanèchite found in Upper Palaeolithic contexts in the region^17^,^18^,^19^,^32^,^33^,^34^.

### Combustion Experiments

Starting from the chemical properties of manganese dioxide, a series of statistically-designed combustion experiments were used to assess whether fire making could be facilitated using wood and either commercial manganese dioxides (coded MD4 to MD6) or powdered material from the Pech-de-l’Azé I *blocs* (coded MD1 to MD3). Mixtures of wood ‘turnings’ and either manganese dioxide or powdered material from Pech-de-l’Azé I *blocs* were either heated or contacted with spark-lit tinder; the effects were monitored on video; thermal imaging camera temperature monitoring and XRD of the residues were used in selected cases (Methods).

When heated on their own, the wood turnings released volatiles and produced a small amount of char but neither the volatiles nor the char ignited and no fire resulted (Supplementary Information 3). Similarly, spark-lit tinder did not ignite the wood. By contrast, mixtures of manganese dioxide with wood ignited, both when heated and when in contact with spark-lit tinder. Ignition produced glowing combustion and, in some cases, small red flames; the volatiles did not ignite and no yellow flames were produced (Fig. 2 and Supplementary Information 3). As little as 6% by weight of manganese dioxide MD6 was sufficient to facilitate combustion. Infrared thermal imaging data showed that whilst the wood turnings did not ignite at 350 ^o^C, the mixtures of wood turnings with manganese dioxide could ignite at temperatures from around 250 ^o^C and sustain combustion over a surprisingly wide range of temperatures (Supplementary Information 4). In identical experiments, powdered material from the Pech-de-l’Azé I *blocs* (MD1, MD2 and MD3) all facilitated the ignition of wood, although one *bloc* (MD1) was somewhat less effective.

XRD analysis of the combustion reaction residues demonstrated that the manganese dioxide’s β-MnO_2_ structure was transformed into the hausmannite structure of Mn_3_O_4_ during combustion, implying the release of oxygen (Fig. 3 and Supplementary Information 2). Based on the infra-red thermal imaging data, the transformation of the manganese dioxide to hausmannite, Mn_3_O_4_ in the combustion experiments, occurred at unexpectedly low temperatures compared with the temperatures normally required for this process^35^,^36^ (see also Supplementary Information 4).

In comparative combustion experiments, the thermally stable oxides, aluminium oxide, titanium oxide and zinc oxide had no beneficial effect on wood combustion; no ignition occurred (Supplementary Information 3). Similarly the barium manganese oxide (romanèchite) had no beneficial effect.

### Thermo-gravimetric Analysis (TGA)

**Figure 1:**
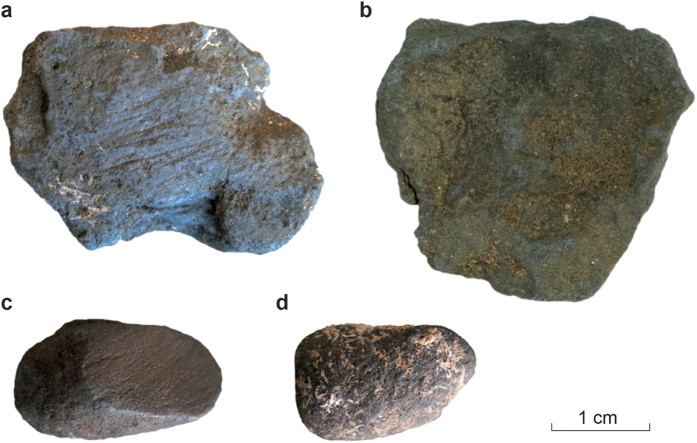
*Blocs* from Pech-de-l’Azé I - both unmodified (b,d) and with abrasion marks (a,c). (**b**) is MD3 from the excavation spoil of early 20th century excavations and the others were recovered in the 2004 and 2005 field work campaigns.

The outcomes of the combustion experiments were validated with the greater control and quantification provided by TGA and differential thermo-gravimetric analysis (DTG). As the temperature is increased in TGA, the beech wood decomposes at temperatures above approximately 220 °C (see the DTG s in Fig. 4a,b and in Supplementary Information 5, Fig. 1). The pyrolysis and volatilisation process^37^ reaches a peak rate at approximately 300°C and the char produced by the pyrolysis^37^ undergoes combustion at temperatures around 460 °C. In the comparative DTG results for manganese dioxide MD4 and wood mixtures (Fig. 4a, 9% and 23% by weight of MD4), the manganese dioxide substantially reduces the char combustion temperature and increases the rate of char combustion sevenfold. Both the wood volatilisation and char combustion occur rapidly at 280 °C to 300 °C, depending on the manganese dioxide content in the mixture with wood turnings. With just 1% by weight of manganese dioxide in the mixture with wood, the volatilisation reactions are not affected but the char’s rate of combustion is significantly increased and the peak rate occurs at a temperature of approximately 370 °C to 380 °C, well below the 460 °C for wood alone (Fig. 4a).

Powdered material from all three Pech-de-l’Azé I *blocs* had an effect on the thermal decomposition of the wood and the combustion of the char in TGA (Supplementary Information 5). *Bloc* MD3 for instance, substantially increased the rate of char combustion and reduced the char combustion peak rate temperature to 300 °C (Fig. 4b). The DTG suggests the temperatures required for initiating combustion with *bloc* MD3 and ‘pure’ manganese dioxide (sample MD4) would be below 300 °C, consistent with the combustion experiment temperatures (Fig. 4, and Supplementary Information 3 and 4).

### Proposed Combustion Mechanism

We propose that the mechanism in facilitating combustion involves the low temperature decomposition of manganese dioxide, stimulated by the reactive gases derived from wood pyrolysis and the consequent release of oxygen that both reduces the critical temperature for ignition and increases the rate of char combustion (Supplementary Information 6). The lack of effect of romanèchite on wood ignition is perhaps explained by the significantly higher temperatures required for the decomposition of romanèchite, the lower amounts of oxygen released^36^ and a reduced effect of wood decomposition volatiles on the decomposition process for romanèchite (see the DTGs for MD7 and MD7 and wood mixtures in Supplementary Information 5).

## Discussion

The composition of the black *blocs* at Pech-de-l’Azé I potentially provides evidence for their probable use. The *blocs* are predominantly manganese dioxide, not romanèchite and the combustion experiments and TGA have shown that only compositions predominantly containing manganese dioxide would be useful in fire-making. Both manganese dioxide and romanèchite would be useful in decoration^32^, although whether either would be preferred for decoration over the less ‘costly’ soot or charcoal is debatable. Whether Neanderthals at Pech-de-l’Azé were simply collecting black *blocs* from one source location or were selecting manganese dioxide in preference to other black materials and from multiple sources is important to our hypothesis that they were deliberately selecting and using manganese dioxide in fire making. Although the quantities and availabilities of different manganese oxides in the Middle Palaeolithic Dordogne region are unknown, there is evidence from both modern sources and from materials collected in the Palaeolithic, for a range of ‘manganese oxide’ materials that were available within reach of Pech-de-l’Azé. Manganese ore outcrops are numerous on the edges of the Massif Central^38^ and whilst most of the regional manganese ores had been extracted by the early twentieth century^32^, an original manganese ore source exists in the limestone within a few kilometres of Pech-de-l’Azé. The source contains traces of both manganese dioxide and romanèchite^32^. Discovery of pyrolusite and romanèchite in a Châtelperronian context at Roc-de-Combe^7^, thirteen kilometres from Pech-de-l’Azé, also indicates that both materials were available to late Middle Palaeolithic Neanderthals. Pyrolusite, romanèchite, todorokite, hollandite and other black manganese oxide ores were all used in the production of Upper Palaeolithic cave wall images in the vicinity, for example at Lascaux, approximately thirty kilometres from Pech-de-l’Azé^19^,^32^,^33^,^34^, implying their availability to Palaeolithic foragers.

Without appropriate data on the variation of ‘manganese oxide’ compositions within and between geological sources in the region, the full implications of the Pech-de-l’Azé I *bloc* compositions for provenance are unknown. Whilst it might be argued that paragenesis might have produced a very variable single source, the relative uniformity of the manganese dioxide content of the *blocs* contrasts with the between-sample variation in arsenic, barium, cobalt and manganite contents and suggests that the *blocs* were not collected from one location. Equally, the availability of a range of ‘manganese oxides’ in the region suggests that the *blocs* were preferentially selected, implying both a capability to recognize the characteristics of these materials - although how this was accomplished is not clear - and an end-use that required the specific properties of manganese dioxide. Pech-de-l’Azé I is not unique and active selection rather than simple collection is supported by the presence of manganese dioxide apparently associated with fire places in the Châtelperronian layers at the Grotte-du-Renne, Arcy-sur-Cure^15^. The black materials said to be of manganese ores at other Mousterian sites (Supplementary Information 1, Table S1) may provide further evidence when the compositions are published.

Our combustion experiments have shown that manganese dioxide promotes the ignition and combustion of wood and that this is not the case with romanèchite. The Pech-de-l’Azé I *blocs* would have had to have been ground to powder for use in facilitating fire lighting and there is archaeological evidence for grinding in the form of a grindstone and abraded *blocs* at Pech-de-l’Azé I^27^ and at Grotte-du-Renne, Arcy-sur-Cure^15^. Spark-lit tinder with manganese dioxide powder is one simple yet effective means of starting wood fires with substantially lower wood auto-ignition temperatures and high rate of combustion. Other methods may be envisaged.

The clear benefits for fire-promotion and the presence of manganese dioxide at Neanderthal sites are not evidence that Neanderthals sourced and used manganese dioxide for fire making purposes nor that they did not use the black material for decorative purposes. However, if different ores have similar decorative properties and Neanderthals selected black manganese oxides that have pronounced oxidizing properties compared to others, we might infer that the choices reflect a fire-related end-use and vice-versa. Chalmin^32^ has shown that specifically for wall ‘painting’, romanèchite produces a more consistent streak than pyrolusite and both are considerably better than manganite; if powdered and dispersed in water, these particular materials are equally effective in decoration. There is apparently no decorative reason for Neanderthals to have favoured manganese oxides over soot and charcoal, or manganese dioxide over other manganese oxides.

In contrast to the “low cost” fire residues, manganese dioxides would have had to have been sourced and transported, at considerably higher costs, which calls for an explanation of such investments outside of body decoration. Our preferred hypothesis is that Neanderthals sourced, selected and transported manganese dioxide for fire making at Pech-de-l’Azé I. Whilst the emphasis here has been on the benefits in fire making, the properties of manganese dioxide could have been exploited in other ways, including improved hafting adhesives^16^.

It is not suggested that manganese dioxide was necessary for fire making or used by Neanderthals all over their geographical range. How Neanderthals developed the innovation is unclear. In fact, the methods of fire production in the Middle Palaeolithic have not been identified^39^ and Neanderthals may only have collected fire from wild fires. However, the fact that fire was used as a tool to produce birch-bark pitch already from the early Middle Palaeolithic onward^40^,^41^,^42^ shows that Neanderthals had the capability to control fire from minimally 200,000 years ago. Such a considerable time depth of fire use would be important to a later recognition of the value of manganese dioxide in fire making.

In reviewing the significance of the Female Cosmetic Coalitions (FCC) model in the context of the European Middle Palaeolithic archaeological record, Power, Sommer and Watts^8^ argue that black “manganese” materials were first present at Pech-de-l’Azé IV and Combe Grenal in the glacial conditions of Marine Isotope Stage (MIS) 4. If analyses shows they are indeed manganese dioxide, these black materials would lend support to an origin in the use of manganese dioxide for fire making in the subsistence challenges of the prolonged cold conditions of MIS 4.

Whilst we can envisage substantial subsistence benefits in the ability to better start, promote and control fire, fire use also comes with a wide range of social benefits and implications^43^. If Neanderthal engagement with materials and processes held subsistence advantages, it may also have been important in the development of complexity in social relationships. Representing fire promotion by manganese dioxide exclusively as a subsistence benefit, no matter how important, risks understating its possible social and symbolic implications^43^,^44^, even though these are notoriously difficult to study in the deep past.

The selection and use of manganese dioxide for fire making is unknown from the ethnographic record of recent hunter gatherers. This unusual behaviour holds potential significance for our understanding of Neanderthal cognitive capabilities through the extent of their knowledge and insights. The actions involved in the preferential selection of a specific, non-combustible material and its use to make fire are not obvious, not intuitive and unlikely to be discovered by repetitive simple trials as might be expected for lithic fracturing, tool forming and tool use. The knowledge and insights suggested by Neanderthal selection of manganese dioxide and use in fire-making are surprising and qualitatively different from the expertise we associate with Neanderthal subsistence patterns from the archaeological record.

We conclude, based on the compositions of the Pech-de-l’Azé I *blocs* and the availability of different black manganese oxides in the Dordogne region, that Neanderthals were preferentially selecting specifically manganese dioxide *blocs*. However manganese dioxide does not have clearly evident advantages in decoration over the carbon-rich materials or the other manganese oxides available to Neanderthals. From the combustion and TGA experiments, it is clear that manganese dioxide is an effective facilitator in fire making, reducing the auto-ignition temperature of wood and substantially increasing the rate of combustion. The archaeological evidence of *bloc* abrasion and grinding stone is consistent with the conversion to powder necessary for use in fire-starting. The intimate association of fire places and manganese dioxide *blocs* at Pech-de-l’Azé I suggest a use in fire making. We hypothesise that fire-making was manganese dioxide’s most beneficial distinguishing attribute available to Neanderthals. Although we should not exclude the possibility that manganese dioxide was used for decoration and social communication, the combustion, compositional and archaeological strands of evidence lead us to the conclusion that late Neanderthals at Pech-de-l’Azé I were using manganese dioxide in fire-making and by implication were producing fire on demand.

## Methods

### Materials

Three commercially available manganese dioxide materials were used in the combustion experiments; two reagent grades from Sigma-Aldrich (product reference 310700, coded MD4 and product reference 217646, coded MD6) and a less pure material supplied by Minerals Water Ltd. (coded MD5). A romanèchite, hydrated barium manganese oxide material (coded MD7) from the Schneeberg mine in Saxony, Germany was also used. Its elemental composition is not inconsistent with romanèchite and the XRD-determined structure has close similarities with a romanèchite XRD reference (Supplementary Information 2). This material may not have had precisely the same properties and behaviour as romanèchite material from the Dordogne region.

Three metal oxides were chosen for comparative experiments, all thermally stable oxides, aluminium oxide, zinc oxide and titanium dioxide. All the oxide materials were reagent-grade materials from the Gorlaeus Laboratorium, University of Leiden. Elemental compositions and crystal structures of the manganese oxides are given in Supplementary Information 2.

Three small blackish coloured *blocs* from the ‘spoil’ of early twentieth century excavations at Pech-de-l’Azé I were studied (coded MD1, MD2 and MD3). These *blocs* were recovered during the 2004–5 fieldwork season led by M. Soressi; they were in the excavation spoil at the entrance of the cave along with artefacts left by previous excavators, mostly in L. Capitan and D. Peyrony’s 1912 excavation. Two were grey-black pebble-like materials and the third (MD3) had a more slab-like appearance with a reddish colour overlying the grey-black material on one side. Each *bloc* was examined by optical and scanning electron microscopy (SEM) with EDX and analyzed by XRD and XRF; approximately two grams in total were used in the combustion experiments.

Ten *blocs* from recorded archaeological contexts in Bordes’ 1970–1 excavations and eleven from Soressi’s 2004–5 excavations were non-destructively analyzed for their XRF compositions and XRD structures. The measured sample set constitutes approximately 5% of the population of *blocs* when MD1 to MD3 are included. The Bordes’ *blocs* appeared to have facets or striations suggesting that they had been deliberately abraded. There were no clearly abraded facets on the eleven *blocs* selected from Soressi’s excavation contexts but there were striations on one *bloc*. The differences confound two variables, recovery location and apparent use, rendering the interpretation of differences more difficult.

The combustible material was untreated beech wood free from bark, converted into turnings using a hand-held electric drill and 22 mm steel bit. Cotton wool and *Ulmus* sp. seed were used as tinder materials.

### Combustion Experiments

**Figure 2:**
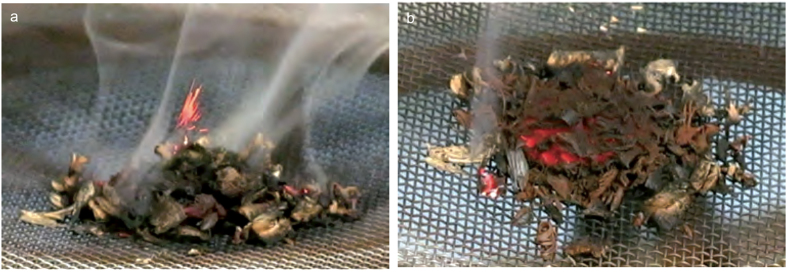
Combustion Experiments with Manganese Dioxide MD4 and Wood Mixtures (a) showing small red flames and volatile emission and (b) the glowing fire combustion phase.

**Figure 3:**
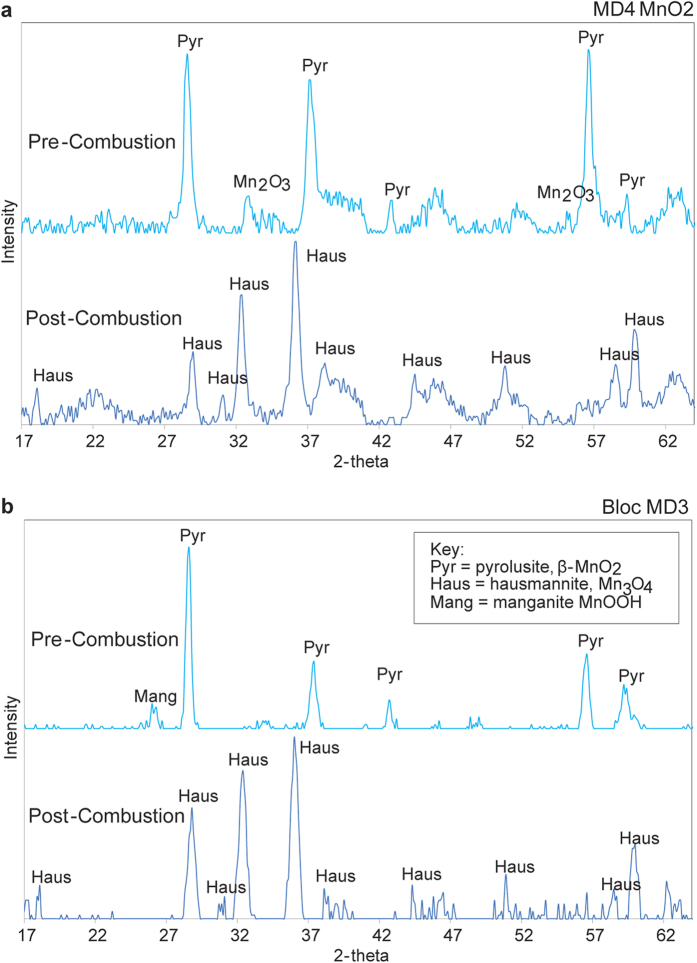
XRD Structures of Manganese Dioxide MD4, *Bloc* MD3 and their Combustion Residues (a) Commercial Manganese dioxide (MD4) and its Combustion Residue and (b). Pech-de-l’Azé I *Bloc* MD3 and its Combustion Residue.

**Figure 4:**
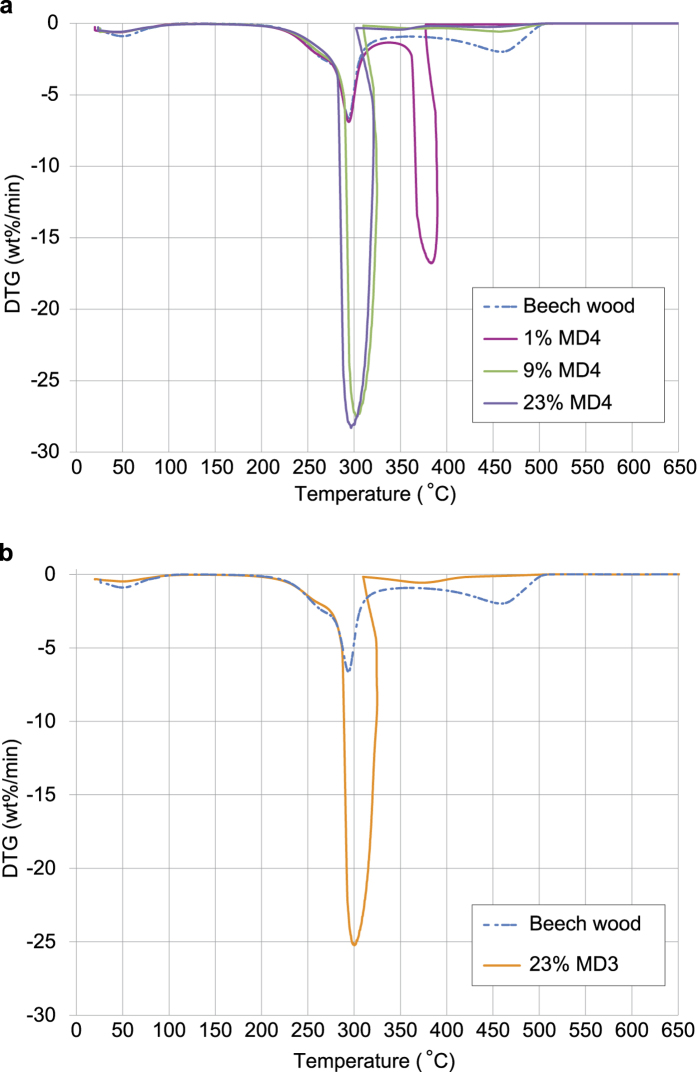
DTG of Wood and Mixtures of Wood and Commercial Manganese Dioxide MD4 and Wood with *Bloc* MD3 (a). DTG of beech wood and Manganese Dioxide MD4 in air and (**b**). DTG of beech wood and Pech-de-l’Azé I *Bloc* MD3 in air.

In the combustion experiments, small amounts of the beech wood turnings (1.5 g) or mixtures of beech (1.5 g) with manganese dioxide (0.1 g to 0.5 g) or powdered materials from the Pech-de-l’Azé I *blocs* or other oxides were placed on a fine steel gauze on a stand within a fume cupboard in a gentle air stream (see Fig. 2). The mixture was heated from below by the flame of a 9.5 cm Sakerhets Tandstickor for fifteen seconds; in some cases the heating time was extended to thirty seconds with a second match. The flame was unable to penetrate the gauze and served to heat the wood via the gauze. For some experiments a Swedish Firesteel 2.0 was used as a source of sparks to light a 0.1 g piece of tinder placed on the surface of the beech turnings. Wherever possible, multiple replication runs were used to validate the outcomes, control runs of beech alone or beech mixed with MD4 or MD6 were used in each phase. In total 120 experimental runs were completed.

The effects were recorded on high definition video. In some experiments the whole combustion process of approximately ten minutes was monitored using either a FLIR A35 or a FLIR T450 thermal imaging camera and combustion temperatures recorded. The temperature data were analyzed using FLIR ResearchIR version 3.4 software (Supplementary Information 4).

### Thermo-gravimetric Analysis Methodology

Thermo-gravimetric differential thermal analysis was performed in nitrogen or air atmospheres using a TA-Instruments SDTQ600. A typical sample mass of 12–15 mg was heated to the desired temperature at a ramp rate of 5 °C/min in a total flow rate of 100 ml/min. Beech wood used for the impregnations was ground and sieved to 90 μm. The ground wood (200 mg) was mixed with manganese dioxide to yield 1% by weight, 9% by weight and 23% by weight of manganese dioxide and wood samples. After addition of manganese dioxide the sample was moistened by 1 ml of de-ionized water mixed and oven dried at 60 °C for five hours.

### X-Ray Fluorescence Spectroscopy (XRF)

Elemental composition (for elements with atomic number greater than 11) was measured using a Thermo Scientific Niton XL3t X-Ray fluorescence (XRF) device with GOLDD detector equipped with a silver anode operating at a maximum of 50 kV and 40 A. Measuring times were 110 seconds. Powder samples were measured with the device inverted and the powder mechanically raised on a scissor-jack table until in full contact with the source/detector window. For *blocs*, the XRF device was mechanically held facing up, with the *blocs* placed above the source/detector window. Measurements were in triplicate, with the samples removed from the source between measurements. The device itself was calibrated by the manufacturer and had been further calibrated by Dr. B. van Os of the Rijksdienst voor het Cultureel Erfgoed (Amersfoort, Netherlands). Quantification of the elements is based on the elements being present as oxides.

### Scanning Electron Microscopy (SEM)

SEM analysis of the fracture surface of *blocs* MD1, MD2 and MD3 was performed using a JSM5910LV equipped with a ThermoScientific SDD detector for energy dispersive X-ray (EDX) analysis. The SEM was operated in low vacuum (30 Pa) at 20 Kv using backscatter electron mode.

### X-ray Diffraction

XRD was performed using a Bruker D8 Discover XRD equipped with a 2D General Area Detector Diffraction System detector. The machine used a copper target and was operated at 40 kV and 30 mA.

## Additional Information

**How to cite this article**: Heyes, P. J. *et al*. Selection and Use of Manganese Dioxide by Neanderthals. *Sci. Rep.* **6**, 22159; doi: 10.1038/srep22159 (2016).

## Supplementary Material

Supplementary Information
