# Supplementary material for: Selection and Use of Manganese Dioxide by Neanderthals
Source: Sci Rep. 2016 Feb 29;6:22159. doi: 10.1038/srep22159 (PMC4770591; doi:10.1038/srep22159)
Supplement: Supplementary Information [file srep22159-s1.pdf]

Supplementary Information

Selection and Use of Manganese Dioxide by Neanderthals

Peter J. Heyes[a], Konstantinos Anastasakis[b], Wiebren de Jong[b], Annelies van Hoesel[a], Wil Roebroeks[a], and Marie Soressi[a*]

[a] Faculty of Archaeology, Leiden University, The Netherlands.
[b] 3mE Process and Energy, Delft University of Technology, The Netherlands.

SI 1 - The Location of Manganese Oxide *Blocs* at Pech-de-l'Azé I and Other Sites in France 2

SI 2 - Compositions and Structures of Materials 5

Combustion Experiment Materials - Composition and Crystal Structure 5

Pech-de-l'Azé I *Blocs* - XRF Composition and XRD Structure 6

Regional 'Manganese Oxide' Compositions 10

Composition and Fire Facilitation 12

Pech-de-l'Azé I *Bloc* Morphology 12

Combustion Experiment Residues 15

SI 3 - Combustion Experiment Outcomes 16

Video Imagery of Selected Combustion Experiments 16

Combustion Experiment Details and Outcomes 18

SI 4 - Infra-Red Thermal Imaging of the Combustion Processes 21

Comparative Combustion Experiments 25

SI 5 - TGA and DTG of Wood or Oxide and Wood Mixtures 27

DTG for Wood in Air and Nitrogen 27

TGA and DTG for Romanèchite MD7 and Wood Mixture 27

TGA and DTG for Manganese Dioxides and Romanèchite (without wood) 28

DTG for Lower Purity Manganese Dioxide (MD5) Mixtures with Wood 29

DTG for Manganese Dioxide MD6 and Wood Mixtures 30

DTG for Pech-de-l'Aze I *Blocs* MD1 and MD2 in Mixtures with Wood 30

TGA and DTG for Iron Oxide and Wood Mixtures 31

SI 6 - Proposed Combustion Mechanism 32

Supplementary References 34

Photographic images in Fig. 1 and Fig. 2 were produced by P.J. Heyes; the images in SI 1 Fig. 1 were produced by M. Soressi; SI 3 Video 1 and Video 2 were created by P.J. Heyes and edited by Jean-Herbert Wzgarda of Société AElementworks. The SEM images in SI 2 Fig. 3 were produced by A. van Hoesel.

Supplementary Information 1 - The Location of Manganese Oxide *Blocs* at Pech-de-l'Azé I and Other Sites in France

SI 1 Figure 1 contains details of the site at Pech-de-l'Azé I. Figure 1a shows a view of the cave entrance, Figure 1b a plane view of the excavations by different excavators, including Bordes (1970-1) and Soressi (2004-5), from which all but three of the *blocs* mentioned in this paper were recovered. Figure 1c shows the locations of the *blocs* and lithic artefacts recovered from the cave. Figure 1d depicts a piece of sandstone with black material on the surface interpreted as the result of grinding *blocs* to powder. SI 1 Table 1 lists several Middle Palaeolithic locations in France from which 'manganese ores' have been recovered.

SI 1 Figure 1 - The Pech-de-l'Azé I Site, Locations of Black *Blocs* and Grindstone (a and b after Soressi et al. 2013 [3])

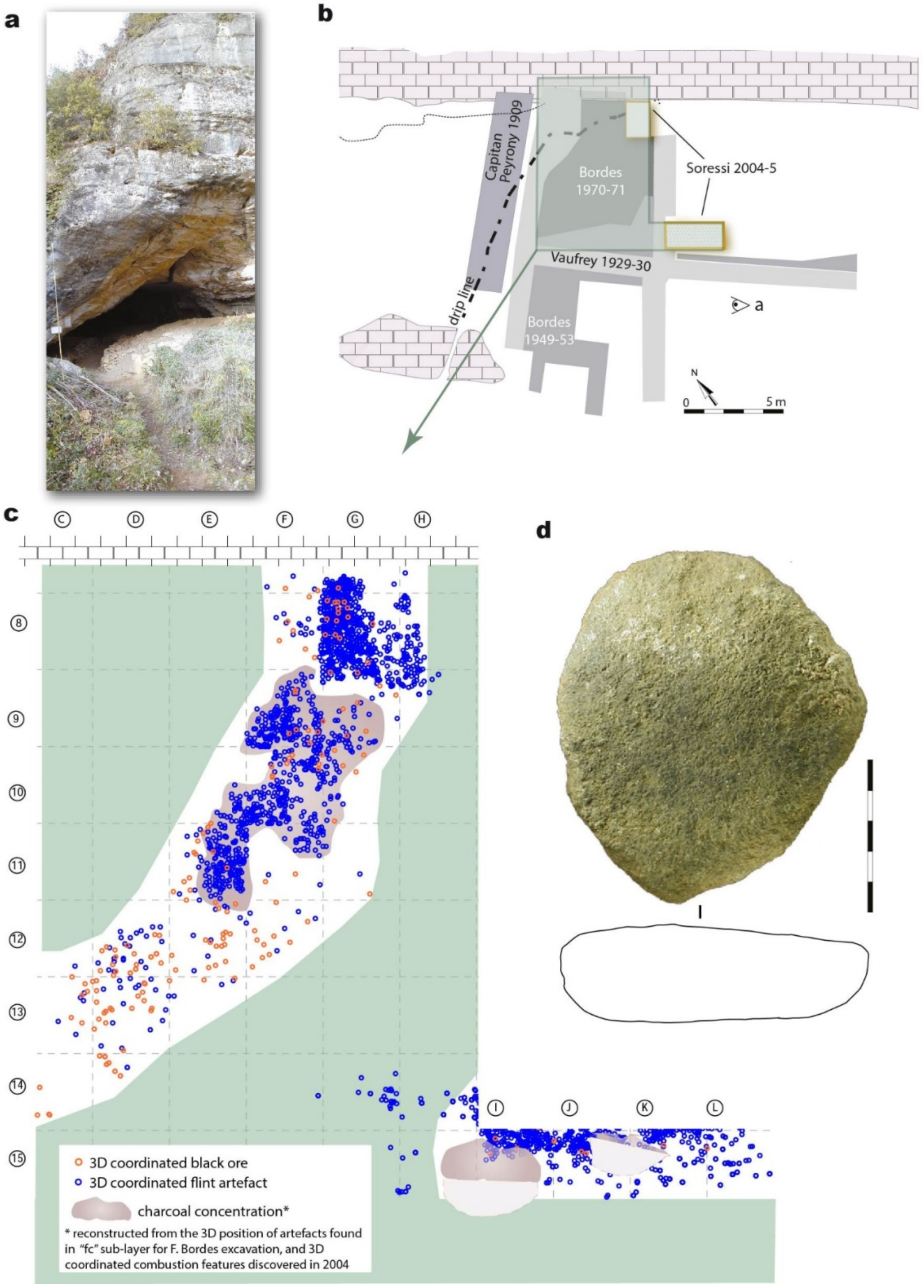

SI 1 Table 1 - Several Mousterian Sites in South West France yielding Manganese Oxide *Blocs* and Evidence for Fire

Site	Level	Culture	Manganese Oxide		Fire		References
			Blocs	**Grindstone**	**Evidence**	**Layer**	
Pech-de-l'Azé I	4, 5, 6, 7	MTA	Yes	Yes (Level 4)	Yes	4, 6, 7	a
Caminade	M3 base, M2	Ferrassie Mousterian	Yes		Yes	M2	b
Pech-de-l'Azé IV	J3a/6A, F1, F3, F4, I1, I2, J1	Typical Mousterian, MTA in F1 to F4	Yes		Yes	6A, possibly F3/3A and I1/4B (heated lithics), 8, 7 through to 4B. Evidence for 4A, 4B and 3A scattered charcoal	c
La Quina	Trench C top		Yes		Yes	8, 6	d
Le Moustier	F, G, H, J	MTA	Yes	Yes	Yes	G-J heated lithics	e
L'Ermitage		MTA	Yes		Yes		f
Combe Grenal	6, 17, 21, 23, 25, 25, 26, 27, 35	Typical Quina, Ferrassie	Yes		Yes	17, 23-25, 27	g

References	Manganese Oxide *Blocs*	Fire
a	1, 2, 3	4, 3, Soressi unpublished
b	2	5
c	2	6, 7, 8
d	9, 10	9, 10
e	11, 12	13
f	14	14
g	2	4, 15, 16

Supplementary Information 2 - Compositions and Structures of Materials

Combustion Experiment Materials - Composition and Crystal Structure

SI2 Table 1 and SI2 Table 2, list the XRF-based composition of the materials used in the combustion experiments and the XRD-based crystal structures. For simplicity, only the main elements present are shown.

SI2 Table 1 : Compositions of Materials used in Combustion Experiments by XRF

Material	Mn	Ca	Al	Ti	Fe	Si	Ba	Co	As	W
MD4	627	0.2	8.1			2.0	0.7	0.1		
MD5	520	0.4	31.4	33.0	0.3	18.7	3.2	0.6		
MD6	607	0.1	7.0	0.4		7.6	1.5	0.2		
MD7	346	0.8					140	5.8	1.2	5.8
All values are in mg/g										

SI2 Table 2 : Structures of Materials used in Combustion Experiments by XRD

Material	Source	Crystal Structure
MD4	Sigma Aldrich	β-MnO_2 , pyrolusite
MD5	Minerals Water	β-MnO_2, pyrolusite
MD6	Sigma Aldrich	No crystalline structure
MD7	Adam Heber, Schneeberg	Probably a romanèchite, $BaMn_5O_{10}.H_2O$

Two of the three manganese dioxide materials used in the combustion experiments (MD4 and MD5) have a β-MnO_2 (pyrolusite) structure the other (MD6) is amorphous. From Supplementary Information 3 it is clear that the crystal structure is not critical to the combustion performance, simply the chemical properties of manganese dioxide. The compositions of the three materials range in MnO_2 content; the theoretical manganese content of pure manganese dioxide is 632mg/g, suggesting that MD4 contains 99%, MD5 82% and MD6 96% of manganese dioxide respectively. The main additional elements are aluminium, titanium, silicon and to a lesser extent barium.

MD7 by contrast has a structure closely similar to a romanèchite XRD reference (SI2 Figure 4); it has the lower manganese content, the high barium content and tungsten (W) content expected of a hydrated barium manganese oxide such as romanèchite from the Schneeberg[17].

SI2 Figure 1 : XRD Comparison of MD7 and Romanèchite References

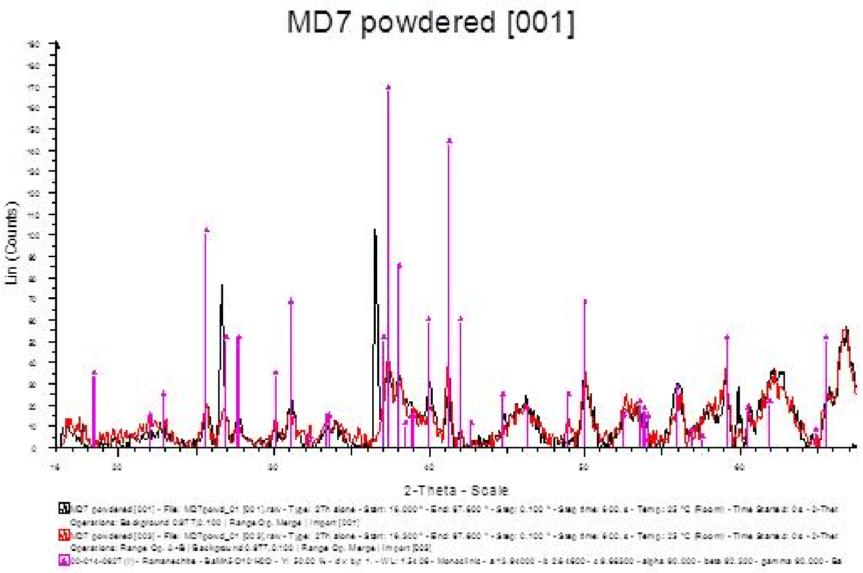

Pech-de-l'Azé I *Blocs* - XRF Composition and XRD Structure

SI2 Table 3, SI2 Table 4 and SI2 Table 5 list the XRF-based composition of the three Pech-de-l'Azé I *blocs* used in the combustion experiments, the ten *blocs* from Bordes excavations and the eleven *blocs* from Soressi's excavations respectively. For simplicity, only the main elements present are shown. SI2 Table 6, SI2 Table 7 and SI2 Table 8 list the corresponding XRD-based crystal structures.

SI2 Table 3 : Compositions of *Blocs* used in Combustion Experiments by XRF

Material	Mn	Ca	Al	Ti	Fe	Si	Ba	Co	As
MD1	563	17.7	6.1	0.1	1.4	21.6	6.4	0.2	0.4
MD2	556	8.7	9.4	0.1	2.3	29.5	3.8	0.3	0.6
MD3	488	11.4	6.0	1.0	20.5	8.3	3.9	2.0	1.8
All values are in mg/g									

SI2 Table 4 : Compositions of *Blocs* from Bordes' Archaeological Contexts by XRF

Bloc	Site Code	Mn	Ca	Al	Ti	Fe	Si	Ba	Co	As
1	PAI-c4-(50)	535	1.4	5.8	0.3	2.1	6.1	2.7	2.6	1.2
2	PAI-c4-D12-410 (93)	532	6.0	5.7	0.3	7.3	8.0	3.3	0.3	1.4
3	PAI-c4-(46)	502	15.5	7.2	0.6	3.4	8.6	9.2	0.4	0.8
4	PAI-c4-F10-1294 (15)	511	8.6	7.4	0.6	3.9	10.7	4.6	0.5	0.4
5	PAI-c4-(43)	510	18.1	6.1	0.6	3.8	8.3	7.6	0.3	1.2
6	PAI-c4-F8-694 (6)	525	13.0	6.5	0.6	4.2	11.0	6.4	0.5	0.6
7	PAI-c4-G9-772 (146)	504	14.4	8.3	0.5	10.6	12.6	3.2	1.0	1.0
8	PAI-c4-E12-1017 (148)	494	5.4	6.7	0.6	21.3	9.1	4.3	0.7	1.9
9	PAI-c4-D12 (99)	515	1.9	5.9	0.4	0.6	7.4	9.2	0.5	0.1
10	PAI-c4-D12-381 (231)	495	7.8	5.2	0.6	6.3	11.9	6.6	0.5	1.7
Values in mg/g										

SI2 Table 5: Compositions of *Blocs* from Soressi's Archaeological Contexts by XRF

Bloc	Site Code	Mn	Ca	Al	Ti	Fe	Si	Ba	Co	As
11	PAI-G8-1169	431	13.0	7.4	1.1	7.6	13.9	13.0	0.8	0.7
12	PAI-L15-14	507	43.3	4.6	0.3	5.6	8.1	4.1	0.2	1.1
13	PAI-G8-1181	503	15.5	6.1	0.3	10.0	11.2	2.9	1.0	0.6
14	PAI-I15-73	336	37.0	3.6	0.6	5.4	12.1	8.0	0.2	0.3
15	PAI-G8-1100	141	121	4.0	1.5	11.2	10.7	39.5	0.5	0.0
16	PAI-G8-873	487	13.4	8.5	0.4	16.4	12.7	4.6	0.5	0.9
17	PAI-I15-79	416	51.1	4.0	0.5	8.3	10.3	10.7	0.9	0.2
18	PAI-G8-458	463	18.9	6.2	0.9	7.5	12.9	9.9	1.0	0.9
19	PAI-I14-376	476	50.8	5.3	0.3	15.0	8.2	3.1	0.2	1.2
20	PAI-I14-328	498	8.4	7.4	0.5	11.7	9.0	3.0	0.2	2.6
21	PAI-H14-3	464	42.5	8.2	0.3	15.7	10.8	3.1	0.1	1.3
Values in mg/g										

SI2 Table 6 Structures of *Blocs* used in the Combustion Experiment by XRD

Bloc	Code	Main XRD	Other Peaks
1	MD1	β-MnO_2, pyrolusite	manganite (MnOOH)
2	MD2	β-MnO_2, pyrolusite	
3	MD3	β-MnO_2, pyrolusite	manganite

SI2 Table 7 : *Bloc* Structures from Bordes' Archaeological Contexts by XRD

Bloc	Site Code	Main XRD Structure	Other Peaks
1	PAI-c4-(50)	β-MnO_2, pyrolusite	
2	PAI-c4-D12-410 (93)	β-MnO_2, pyrolusite	
3	PAI-c4-(46)	β-MnO_2, pyrolusite	
4	PAI-c4-F10-1294 (15)	β-MnO_2, pyrolusite	manganite
5	PAI-c4-(43)	β-MnO_2, pyrolusite	
6	PAI-c4-F8-694 (6)	β-MnO_2, pyrolusite	manganite
7	PAI-c4-G9-772 (146)	β-MnO_2, pyrolusite	manganite
8	PAI-c4-E12-1017 (148)	β-MnO_2, pyrolusite	manganite
9	PAI-c4-D12 (99)	β-MnO_2, pyrolusite	
10	PAI-c4-D12-381 (231)	β-MnO_2, pyrolusite	manganite

SI2 Table 8 : *Bloc* Structures from Soressi's Archaeological Contexts by XRD

Bloc	Site Code	Main XRD Structure	Other Peaks
11	PAI-G8-1169	β-MnO_2, pyrolusite	manganite
12	PAI-L15-14	β-MnO_2, pyrolusite	manganite
13	PAI-G8-1181	β-MnO_2, pyrolusite	manganite, possibly hollandite?
14	PAI-I15-73	almost no signal	possibly pyrolusite, manganite?
15	PAI-G8-1100	β-MnO_2, pyrolusite	manganite
16	PAI-G8-873	β-MnO_2, pyrolusite	manganite
17	PAI-I15-79	very bad signal	possibly manganite
18	PAI-G8-458	β-MnO_2, pyrolusite	manganite
19	PAI-I14-376	β-MnO_2, pyrolusite	manganite
20	PAI-I14-328	β-MnO_2, pyrolusite	
21	PAI-H14-3	β-MnO_2, pyrolusite	manganite

XRF analysis of the surface of the twenty one *blocs* from archaeological contexts shows that all except two (PAI-I15-73 and PAI-G8-1100) have high levels of manganese. XRD indicates that nineteen of the *blocs* have a β-MnO_2 (pyrolusite) structure that is consistent with a composition dominated by manganese dioxide. Thirteen *blocs* contain some manganite (ϒ-MnOOH). For two *blocs* (PAI-I15-73 and PAI-I15-79) the XRD signal is too poor to determine the crystal structure, if any. Of the two lower manganese content *blocs*, there was substantial variation in the measured manganese content of PAI-I15-73 such that the content was not significantly different to two other *blocs* in two sample hypothesis testing. The variability limits any interpretation of the significance of PAI-I15-73.

Manganite is the only other manganese oxide identified with confidence. Unlike other complex manganese oxides, manganite decomposes to manganese dioxide at approximately 300°C [18] and would be expected to contribute to the fire making behaviour discussed in the combustion mechanism.

All the *blocs* have varying contents of other elements, principally calcium, iron, silicon and aluminium. All contain only small amounts of barium except one, PAI-G8-1100, which has a relatively high barium content but also a very high calcium content and a low manganese content (SI3 Table 5). However the

barium content of PAI-G8-1100 is substantially less than expected in barium manganese oxide, romanèchite[17] and measured in romanèchite sample MD7 (SI3 Table 1); the XRD pattern shows that PAI-G8-1100 contains β-MnO_2 (pyrolusite) and not romanèchite. None of the *blocs* have an XRD pattern suggestive of romanèchite. The calcium content of PAI-G8-1100 is statistically higher than other *blocs*, including *blocs* from the same location, G8, suggesting the calcium may be from a limestone environment at its source location, not Pech-de-l'Azé I, although the absence of evidence for the compositions and variability in the original sources limits the conclusions that can be drawn.

There is compositional variation both between samples and within samples; the variations are not consistent, in other words a sample with an unusual value in one element may be within the range of variation in other elements. The variation in the calcium contents suggests a varying presence of surface contamination from either a limestone source location or the cave environment.

There is substantial variation in the iron, silicon and aluminium contents but this variability may be associated with surface contamination on these unwashed samples. Elements such as barium, cobalt and arsenic are more likely to be intrinsically associated with the manganese dioxide and their variation may suggest whether the samples were from different locations. SI3 Figures 2a, 2b and 2c show the means and standard deviations for barium, cobalt and arsenic contents respectively in the *blocs*. Two sample hypothesis testing using Statgraphics version 5.1, shows that there are statistically significant differences in composition between *blocs* for each of the three elements[19]. This pattern of variation is also supported by the differences in manganite content and crystallinity in SI3 Tables 7 and 8.

SI2 Figure 2a : Barium Content in *Blocs* (error bars are one standard deviation)

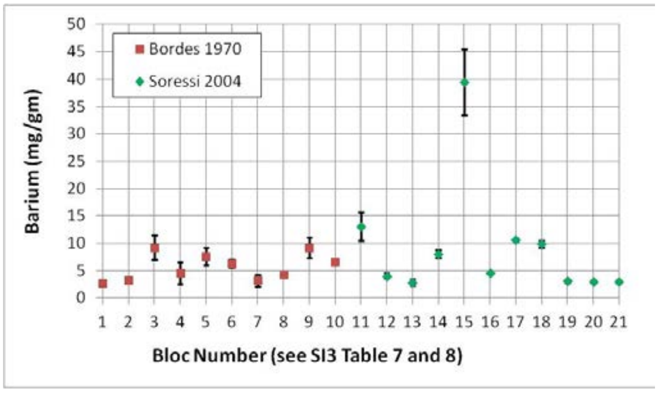

SI2 Figure 2b : Cobalt Content in *Blocs* (error bars are one standard deviation)

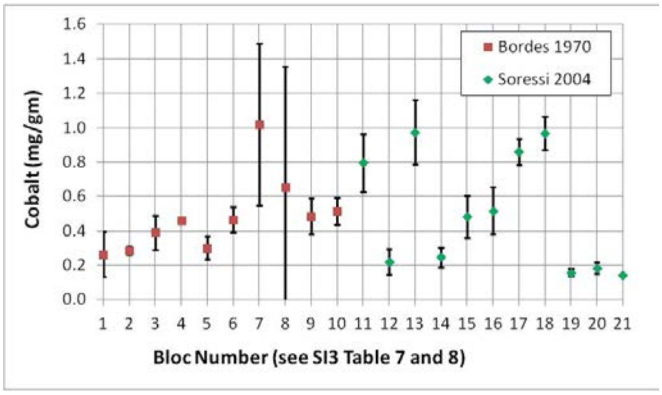

SI2 Figure 2c : Arsenic Content in *Blocs* (error bars are one standard deviation)

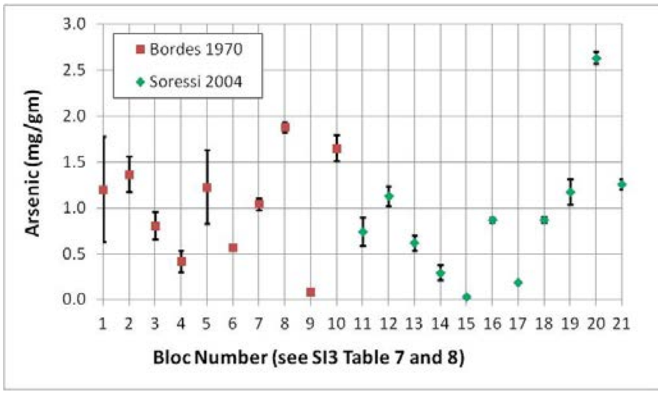

Regional 'Manganese Oxide' Compositions

Without information on the variation of 'manganese oxide' compositions in the regional geological sources , the full implications of the statistically significant differences in *bloc* compositions for provenance are unknown. Whilst the significant differences suggest there were several sources for the manganese dioxide *blocs*, information on the variation in the sources is required for the context of the between and within sample variation.

The compositional variation of the Palaeolithic sources of manganese oxides is unknown. Most of the regional manganese ores had been extracted by the early twentieth century[20] and any compositional data that may have been gathered are not available. The compositions of materials from two sources of manganese ores close to Pech-de-l'Azé (Le Theil and Pech de Bord) and one near Lascaux (La Pagésie) have been reported by Chalmin[20]. Le Theil and La Pagésie comprise the residues of two depleted geological sources; Pech de Bord is not a geological source but a mound of collected manganese ore material and so perhaps less useful. Comparison of their compositions with the Pech-de-l'Azé I materials might provide limited context for the Pech-de-l'Azé I *blocs*. Chalmin[20] comments on the nature of the materials as follows:

"*Les blocs noirs ramassés dans le gisement de La Pagésie sont constitués principalement d'une phase de quartz, de calcite, de goethite, de romanéchite et d'une phase minoritaire pyrolusite. Concernant les blocs du Theil, la phase majoritaire est constituée de quartz et de calcite et l'oxyde de manganèse très minoritaire est une phase de romanéchite. Le gisement du Pech de Bord est plus riche en oxyde de manganèse principalement sous forme de romanéchite, mais on note aussi la présence de pyrolusite avec un peu de calcite et du quartz*".

Chalmin's compositional data[20] appear to be from multiple measurements of one sample from each source using Proton-Induced X-ray Emission. If so the compositional variation does not represent the variation in the source, solely the microscopic inhomogeneity in the samples.

Chalmin[20] cites multiple measurements for seven elements (Al, Si, K, Ca, Mn, Fe, Ba) in materials from Le Theil, Pech de Bord and La Pagésie that permit comparison with elemental concentrations in the Pech-de-l'Azé I materials. Based on two sample hypothesis testing, the elemental concentrations from the three sources differ significantly from the mean values of the Pech-de-l'Azé I *blocs* in all cases, with the exceptions of potassium in La Pagésie and iron in Pech de Bord (note that one *bloc* PAI-G8-1100 was excluded because it differs somewhat from the rest of the *bloc* population). When the concentrations of ten elements (Al, Si, K, Ca, Mn, Fe, Ba, Co, As and Ti) are analyzed, in only 20% of cases is the elemental concentration from the three source materials within the range from 50% of the mean concentration to 200% of the mean concentration of the samples from the Pech-de-l'Azé I *blocs* (excluding PAI-G81100). It is noteworthy that the calcium contents of the materials from Le Theil, Pech de Bord and La Pagésie are substantially greater than in the *blocs* from Pech-de-l'Azé I and the manganese concentration substantially lower. Importantly, both the elemental compositions and the presence of romanèchite in the materials from the geological sources distinguish these materials from the *blocs* at Pech-de-l'Azé I where no romanèchite was detected. The *blocs* were not a random sample from materials available in the region.

In the absence of comparable information on source to source and within source composition variability, it is difficult to relate the *bloc* variation to particular common sources and conclude whether more than one source was exploited. Whilst it might be argued that paragenesis has led to variability in the manganese oxidation state and the presence of manganite in some samples, the significant differences in individual elements such as barium, cobalt and arsenic suggest that the *blocs* did not come from a common source but from a range of sources.

Composition and Fire Facilitation

With the possible exception of the silicon content of MD1 and MD2 (single measurements), the compositions of the three *blocs* from the excavation spoil used in the combustion experiments, fall within the compositional population of the samples from archaeological contexts even when PAI-G8-1100 is excluded[19]. In this case we infer that the materials from archaeological contexts would behave in a manner similar to *blocs* MD1, MD2 and MD3 in combustion, with the possible exception sample PAI-G8-1100. The low manganese content and high barium content of PAI-G8-1100 might well affect its behaviour and should be tested as part of a programme to assess the effect of composition on fire making performance. The compositional reasons for the differences between the combustion performances of *blocs* MD1 and MD3 are not certain but may relate to the presence of particular minor elements (SI2 Table 3) that might inhibit the reducing atmosphere decomposition process.

Pech-de-l'Azé I *Bloc* Morphology

The three *blocs* from the excavation spoil (MD1, MD2 and MD3) used in the combustion experiments were analyzed in more detail using SEM and EDX (SI2 Figures 3 and 4).

SI2 Figure 3 : SEM Images of the Fracture Surfaces of MD1 (a), MD2 (b) and MD3 (c, d, e and f).

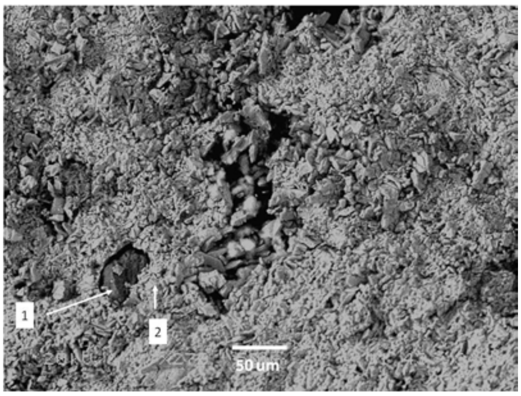

a *Bloc* MD1

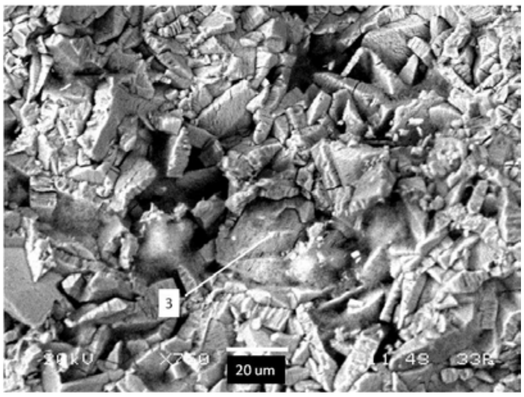

b *Bloc* MD2

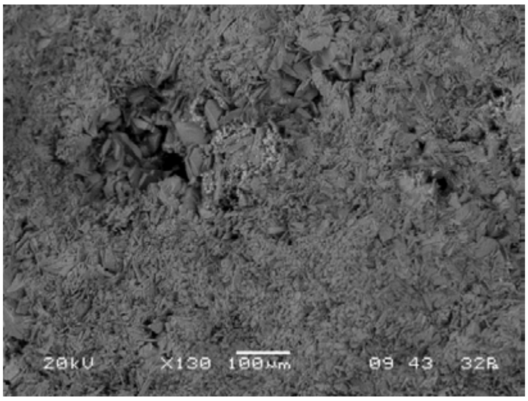

c *Bloc* MD3

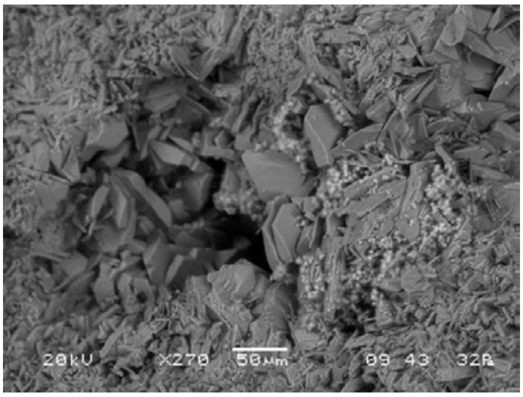

d *Bloc* MD3 at higher magnification

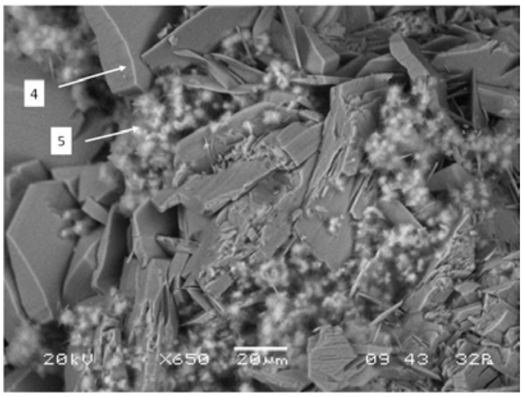

e *Bloc* MD3 crystals and high barium content

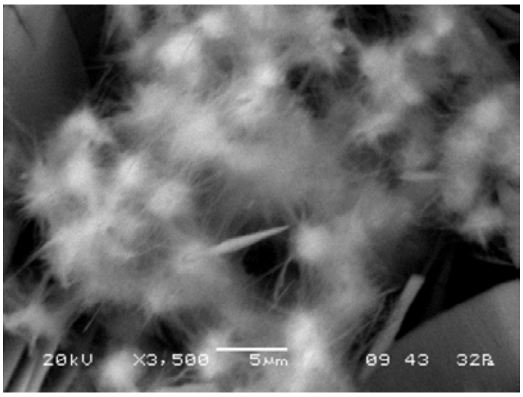

f *Bloc* MD3 high barium content features

SI2 Figure 4 : EDX of Fracture Surfaces of *Blocs* MD1, MD2 and MD3

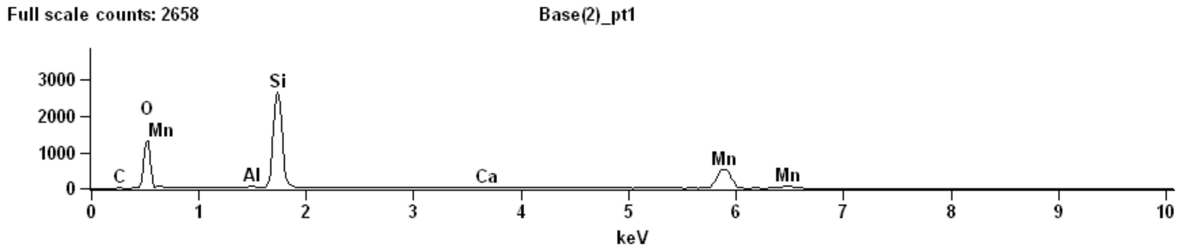

3a - MD1 at Location 1 in SI3 Figure 3a

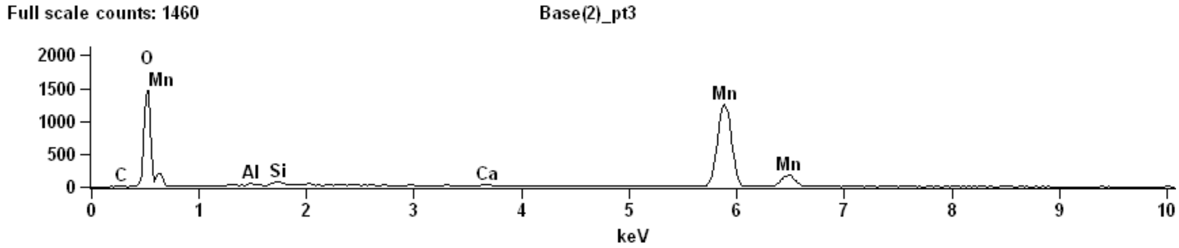

3b - MD1 at Location 2 in SI3 Figure 3a

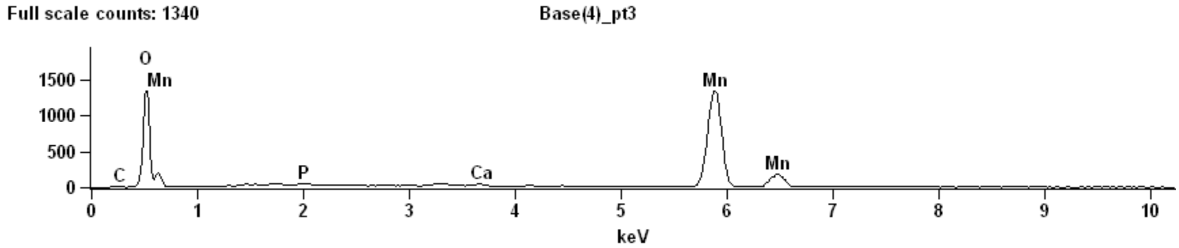

3c - MD2 at Location 3 in SI3 Figure 3b

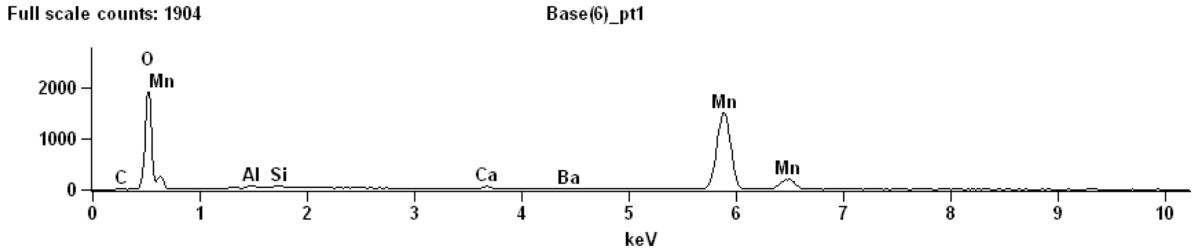

3d - MD3 at Location 4 in SI3 Figure 3e

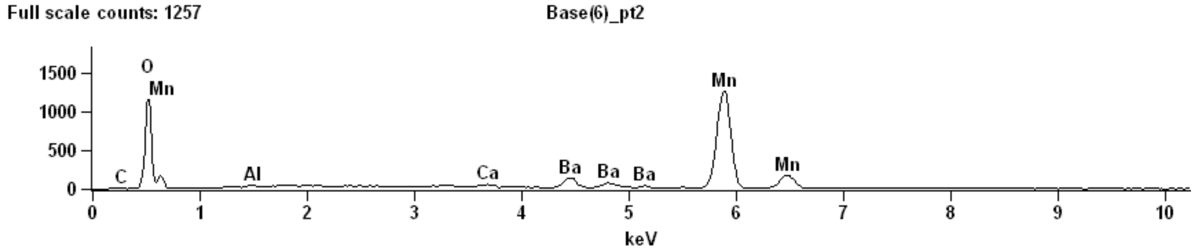

3e - MD3 at Location 5 in SI3 Figure 3e

SEM imaging of the fracture surface of these *blocs* shows that the *blocs* mainly consist of randomly oriented, flat (<10 µm thick) manganese oxide crystals (SI2 Figure 3). Some of the manganese oxide crystals contain fractures (SI2 Figure 3b), most obviously in MD2. *Bloc* MD1 contained a few quartz grains (<50 µm) incorporated within the manganese oxide matrix. EDX analysis (SI2 Figure 4) shows that the barium observed in the XRF is not part of the bulk material of the *blocs*. Rather than being incorporated in the manganese oxide crystals, the barium is present as a separate material in some of the spaces between the manganese oxide crystals. This barium-rich material is observed in the SEM images as groups of bright 'cotton candy ball' feature (SI2 Figure 3f).

<u>Combustion Experiment Residues</u>

SI2 Table 9 lists the XRD analyses of residues from several different combustion experiments described in Supplementary Information 3. All the manganese dioxide containing samples, MD1 to MD6, transformed to hausmannite, Mn_3O_4.

SI2 Table 9 : Manganese Oxide Structures in Combustion Reaction Residues by XRD

Initial Material	Run Code	XRD Structure	Residue	Note
MD1	088	Hausmannite	Mn_3O_4	MnO_2 reduced to Mn_3O_4
MD2	089	Hausmannite	Mn_3O_4	MnO_2 reduced to Mn_3O_4
MD3	090	Hausmannite	Mn_3O_4	MnO_2 reduced to Mn_3O_4
MD4	063	Hausmannite	Mn_3O_4	MnO_2 reduced to Mn_3O_4
MD5	093	Hausmannite	Mn_3O_4	MnO_2 reduced to Mn_3O_4
MD6	066, 086	Hausmannite	Mn_3O_4	MnO_2 reduced to Mn_3O_4

Supplementary Information 3 - Combustion Experiment Outcomes

Video Imagery of Selected Combustion Experiments

The following links provide access to two videos. A first video showing three selected combustion experiment runs: (a) beech wood turnings alone, (b) beech turnings and manganese dioxide code MD4, (c) beech turnings and powdered material from Pech-de-l'Azé I *bloc* MD3 (SI3 Video 1). A second video is of temperature data from the thermal imaging camera monitoring the combustion of a mixture of beech turnings and manganese dioxide MD6 (SI3 Video 2). Each video begins with the fifteen second heating stage and follows the active combustion phase for approximately three minutes of the nine minutes typical for the combustion of 1.5g of wood turnings with manganese dioxide ; only the thermal image video continues to the completion of combustion. For wood turnings alone there is no active combustion phase and the video terminates after emissions cease. None show the initiation of combustion with spark-lit tinder.

In the case of the beech turnings alone, no combustion occurs, simply the emission of volatiles as the heated material undergoes thermal decomposition of the hemi-cellulose and cellulose in contact with the hot gauze. These cease shortly after the source of heat is removed. The experiment run with manganese dioxide MD4 and beech turnings shows the active combustion stimulated by contact with the hot gauze through to the establishment of a glowing fire. Some small red flames are visible but the volatile emissions do not ignite to produce the yellow flames of a typical early stage wood fire. The video for Pech-de-l'Azé I *bloc* MD3 demonstrates the active combustion through to the glowing fire phase but without any visible red flames. The thermal imaging video shows the temperatures of the steel gauze and across the mixture throughout the combustion of a manganese dioxide MD6 and wood turnings mixture. The software automatically adjusts the colour-based temperature scale on the right hand side; the temperature indicated by a particular colour changes and both the scale and image need to be viewed closely to understand the temperature changes. Further information on this experimental run is presented in Supplementary Information 4.

SI 3 Video 1: Three selected combustion experiment runs (a) beech wood turnings alone, (b) beech turnings and manganese dioxide coded MD4, (c) beech turnings and powdered material from Pech-de-l'Azé I *bloc* MD3.

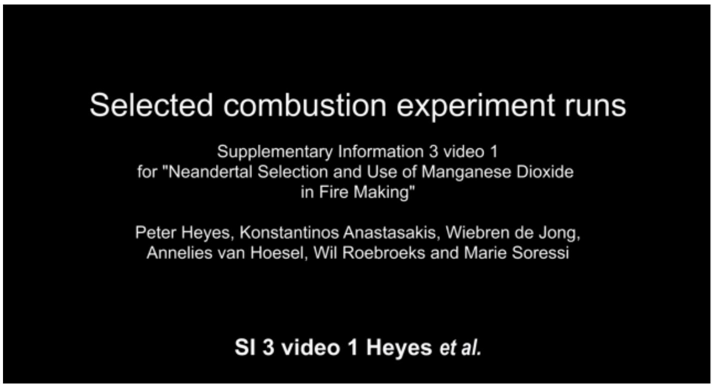

Click here to read the video

(https://www.youtube.com/watch?v=0CvVq3TstDE)

SI 3 Video 2: Temperature data from the thermal imaging camera monitoring the combustion of a mixture of beech turnings and manganese dioxide coded MD6.

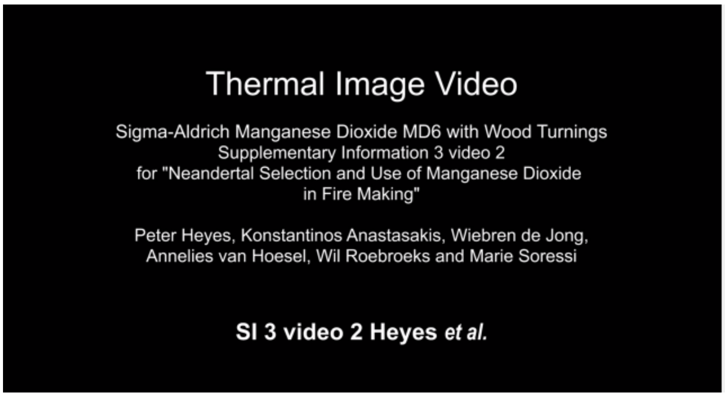

Click here to read the video

(https://www.youtube.com/watch?v=-UTOZFJSNFw)

Combustion Experiment Details and Outcomes

Two tables, SI3 Table 1 and SI3 Table 2 contain summary details of selected combustion experiments. For SI3 Table 1, beech wood turnings or mixtures of beech wood turnings and different oxide powders were heated for fifteen or thirty seconds through a metal gauze by a match flame. The flame itself did not contact the wood or wood/oxide mixtures. The outcomes are shown in the table.

In every case the wood on its own failed to ignite but produced smoke and volatile organic emissions whilst heated and left a small amount of char on the gauze. Extending the heating time from fifteen to thirty seconds had no additional effect. The manganese dioxides MD4, MD5 and MD6 all consistently caused the wood to ignite and combust as a glowing red fire; small red flames were seen with MD4 and MD6. The volatile emissions did not ignite or produce the yellow flames typical of an early stage wood fire. The amount of manganese dioxide required for ignition was as little as 0.1g with 1.5g of wood. The mixtures of wood and the thermally stable oxides aluminium oxide, zinc oxide and titanium dioxide did not ignite.

Mixtures of wood and powdered material from the three Pech-de-l'Azé I *blocs* MD1, MD2 and MD3, all ignited although MD1 required the longer heating time. There was too little material for repeat runs except with MD3 where the effect was seen to be consistent.

The effects of lit tinder in contact with a mixture of wood turnings and different oxides are shown in SI3 Table 2. Whilst the tinder materials, cotton wool or elm seed burned completely, they did not ignite the wood turnings. Mixtures of wood turnings and manganese dioxides were consistently ignited by the tinder and produced glowing fire combustion. The thermally stable oxides aluminium oxide, zinc oxide and titanium dioxide had no effect; their mixtures with wood turnings did not ignite.

Information on combustion temperatures is presented in Supplementary Information 4.

S3 Table 1 : Effects of Manganese Dioxides on the Ignition and Combustion of Beech Turnings using Heated Mixtures

№	Combustible		Oxide		Heating	Burn	Outcome	Run Code
	Material	(g)	Material	(g)	(s)			
1	Beech	1.5	None		15	No	Smoke issued only when heated, some char on the gauze.	009, 060, 070, 036, 053, 091
2	Beech	1.5	None		30	No	Smoke issued only when heated, some char on the gauze.	106, 107
3	Beech	1.5	MD6	0.3	15	Yes	Small red flames, sparks, smoke, material glowing reddish throughout.	010
4	Beech	1.5	MD6	0.5	15	Yes	Small red flames, sparks, smoke, material glowing reddish throughout.	011, 066, 084, 040, 046, 062, 084, 092, 094
5	Beech	1.5	MD4	0.5	15	Yes	Small red flames, sparks, smoke, material glowing reddish throughout.	038, 047, 063, 96
6	Beech	1.5	MD4	0.3	15	Yes	Smoke emitted continuously, material glowing reddish throughout.	117
7	Beech	1.5	MD4	0.2	15	Yes	Tiny red flames, smoke, material glowing reddish throughout.	118
8	Beech	1.5	MD4	0.1	15	Yes	Smoke in increasing amounts, material glowing reddish throughout.	119, 120
9	Beech	1.5	MD5	0.5	15	Yes	Smoke, material glowing reddish throughout, no flames or sparks.	029, 048, 064, 093
10	Beech	1.5	MD7	0.5	15	No	Smoke profuse but only whilst heated, no combustion	116
11	Beech	1.5	MD7	0.5	30	No	Slight char of the wood but no combustion	115
12	Beech	1.5	Al2O3	0.5	15	No	Smoke when heated	097
13	Beech	1.5	TiO2	0.5	15	No	Smoke when heated	101
14	Beech	1.5	ZnO	0.5	15	No	Smoke when heated	099
15	Beech	1.5	MD1	0.5	15	No	Smoke profuse but only whilst heated.	087
16	Beech	1.5	MD1	0.5	30	Yes	Smoke continuous, glowing red.	088
17	Beech	1.5	MD2	0.5	15	Yes	Smoke but not profuse, some glowing red, slowly spreading.	089
18	Beech	1.5	MD3	0.5	15	Yes	Smoke locally profuse, glowing red.	076, 077, 090

Notes

1 MD4 contains 99%, MD5 82% and MD6 96% manganese dioxide respectively based on XRF measurements
2 MD7 is a hydrated barium manganese oxide, romanèchite
3 Al2O3 is aluminium oxide, TiO2 titanium dioxide and ZnO zinc oxide
4 MD1, MD2 and MD3 are powder from *bloc*s from Pech-de-l'Azé I

SI3 Table 2 : Effects of Manganese Dioxides on the Ignition and Combustion of Beech Turnings using Spark-lit Tinder

№	Combustible		Oxide		Tinder	Burn	Outcome	Run Code
	Material	(g)	Material	(g)				
1	None		None		CW		Burned for 20s to 30s.	054, 057
2	Beech	1.5	None		CW	No	CW burned. Char on surface fragments over a small area.	043, 055, 056, 107
3	Beech	1.5	MD6	0.5	CW	Yes	Small red flames, smoke, material glowing reddish throughout.	044, 067
4	Beech	1.5	MD4	0.5	CW	Yes	Small red flames, smoke, material glowing reddish throughout.	045, 105
5	None		None		Elm		Burned for 10s.	
6	Beech	1.5	MD4	0.5	CW	Yes	Small red flames, sparks, material glowing reddish	105
7	Beech	1.5	Al2O3	0.5	CW	No	CW burned, slight char on some surface fragments	098
8	Beech	1.5	TiO2	0.5	CW	No	CW burned, slight char on some surface fragments	102
9	Beech	1.5	ZnO	0.5	CW	No	CW burned, slight char on some surface fragments	100
10	Beech	1.5	Ochre	0.5	CW	No	CW burned, slight char on some surface fragments	104

Notes

1 CW is 0.1g cotton wool
2 Elm is 0.1g *Ulmus* sp. seed
3 MD4 contains 99%, and MD6 96% manganese dioxide respectively based on XRF measurements
4 Al2O3 is aluminium oxide, TiO2 titanium dioxide and ZnO zinc oxide

Supplementary Information 4 - Infra-Red Thermal Imaging of the Combustion Processes

The FLIR A35 thermal imaging camera recorded temperatures over the nine minutes of the combustion process for MD6 manganese dioxide mixed with beech turnings, in the weight ratio 1 to 3. The data is presented in two forms; charts of time against temperature and charts of the temperature distribution across the combustion. The time-temperature charts are in the form of a single spot on the gauze and three areas of the combustible material. The temperature charts are valuable in conjunction with TGA in deducing the thermal processes involved in manganese dioxide-promoted combustion. One limitation is that they represent the surface temperatures of the spot or area selected, not necessarily the temperatures within the combustible material.

Within the temperature distributions, three elliptical areas El1, El2 and El3 and one spot location SP3 were selected for temperature plots against time (locations are marked in SI4 Figure 1). SP3 is on the gauze close to the first brief initiation of combustion at El3. El2 is the area of coalescence of small combustion spots and the initiation of the continuing combustion process throughout the material. El1 is the area active in the main phase of combustion by spread from El2.

Temperatures against time at El1, El2, El3 and SP3 are shown in SI4 Figures 3 to 8; SI4 Figures 1 and 2 show the temperature distributions at initiation and after 197 seconds of combustion, respectively.

The combustion was apparently initiated by gauze temperatures around 200°C (SI4 Figure 3) although this may be an underestimate depending on how close point SP3 was to the initiation. The combustion temperature at El3 rose very rapidly, in less than 50 milliseconds, to values above 400°C (SI4 Figure 4). The initial high temperature combustion at El3 lasted for less than two seconds; the temperature dropped rapidly to around 150°C before rising more gradually to 550°C after 30s (SI4 Figure 5). The process again changed as the temperature rapidly fell to 150°C after 40s for about 10s. After 50s elapsed time, temperatures rose to 400°C (SI4 Figure 6), rising to 600°C before settling into the range 550 to 600°C, with a glowing red appearance until all the beech material had been consumed after nine minutes. Combustion spread to the adjacent areas El1 and El2 and the timing of the temperature decay as combustion concluded depended on the masses of material available in specific areas (SI4 Figures 7 and 8).

Combustion occurred at 400°C in the early stages but was apparently capable of being sustained for brief periods at temperatures below 200°C. The brief very low temperature combustion is both unusual and unexpected and may be significant in fire making facilitation. Combustion was later maintained as a glowing fire largely without visible gaseous emissions in the range 550 to 650°C.

SI4 Figure 1

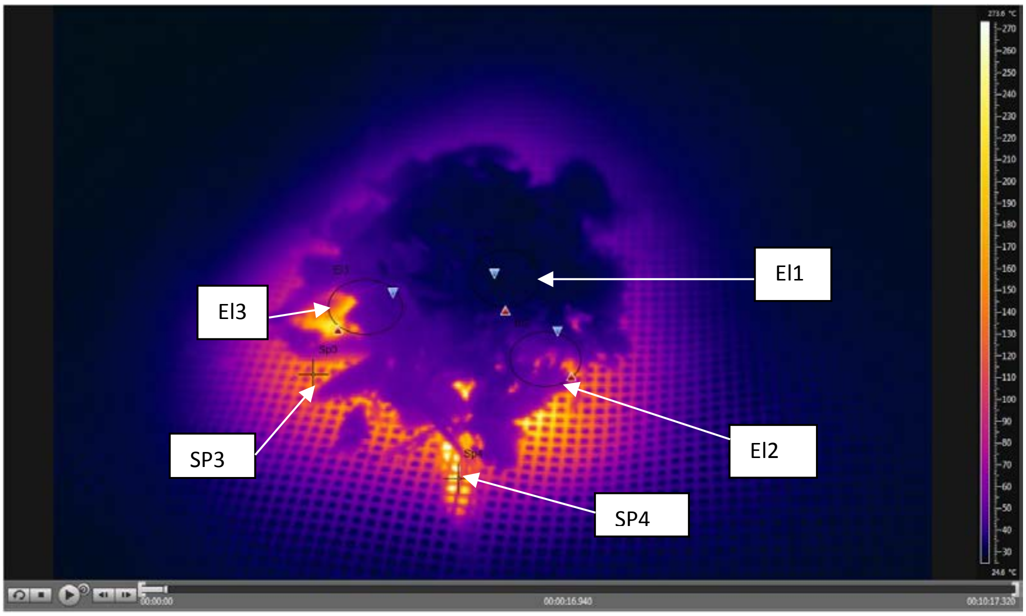

Time 17s - Gauze being heated by the flame, El3 in combustion at 330°C

SI4 Figure 2

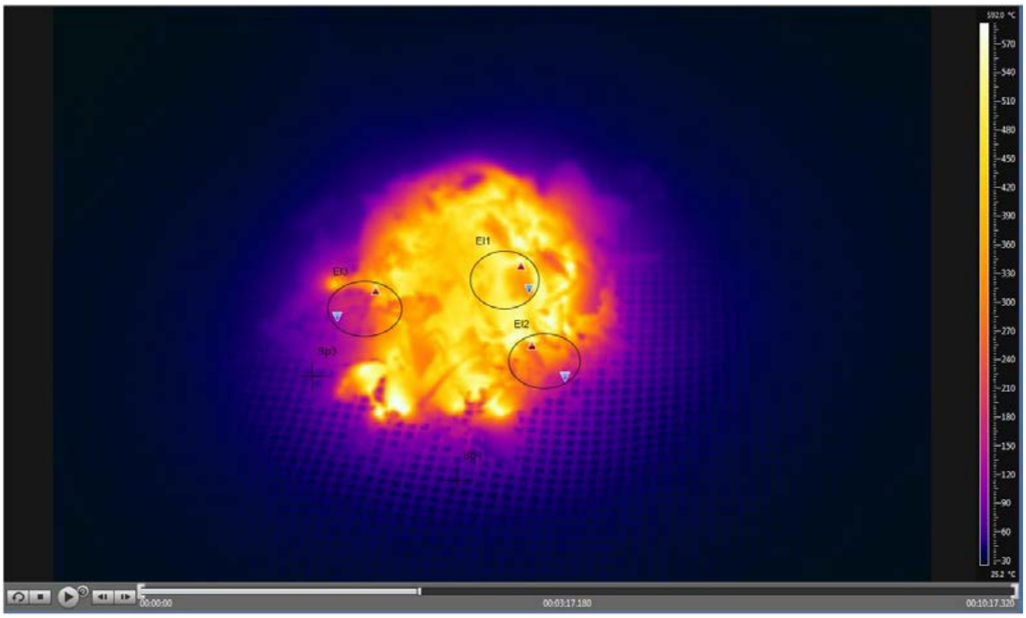

Time 197s - Combustion area at maximum, El1 at 570-590°C

SI4 Figure 3 : SP3 Gauze Temperature Close to the Initiation Point

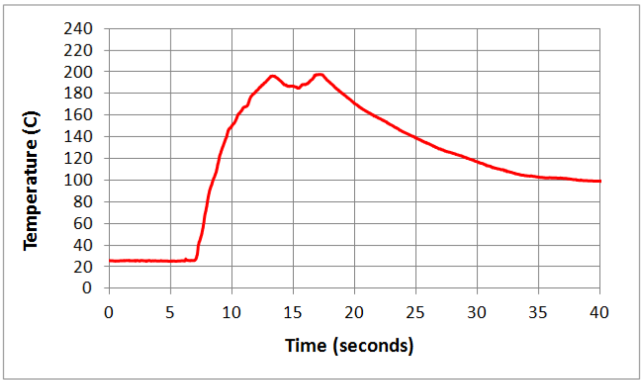

SI4 Figure 4 : EI3 Temperature of Combustion Initiation (6 to 8s)

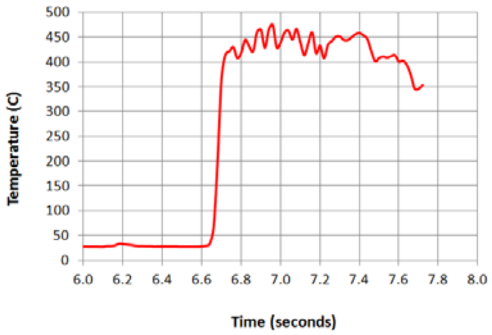

SI4 Figure 5 : EI3 Temperature of Combustion Initiation (0 to 40s)

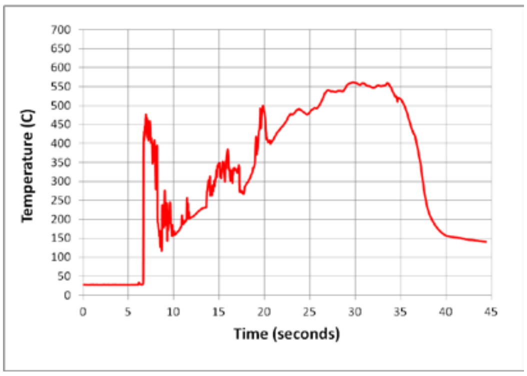

SI 4 Figure 6 : El3 Temperature (0 to 620s)

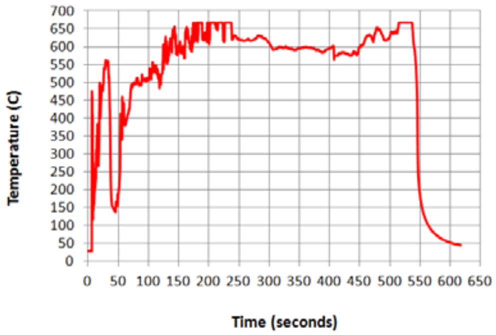

SI4 Figure 7: El2 Temperature (0-620s)

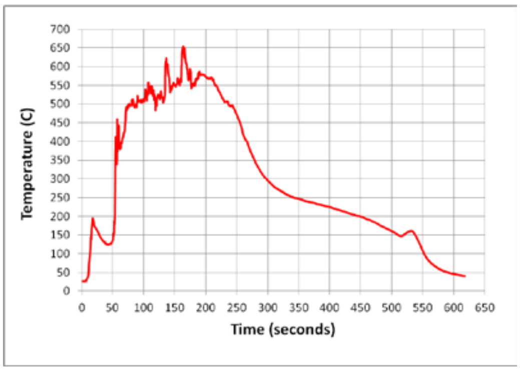

SI 4 Figure 8 : El1 Temperature of the Central Area (0 to 620s)

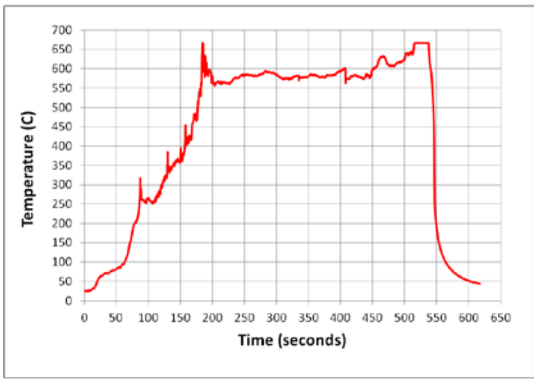

Comparative Combustion Experiments

The combustion temperatures experienced with mixtures of wood and Pech-de-l'Azé I *blocs* MD1, MD2 and MD3, manganese dioxides MD4, MD5 and MD6 and wood alone were measured at four locations, SP1, SP2, SP3 and SP4 using the FLIR T450 camera and FLIR software. SP1 was the gauze temperature close to the start of combustion and SP2 to SP4 points on the mixture at increasing distances from SP1. The charts for each material are shown in SI4 Figure 9a to 9h. In general, the gauze reached 350°C to 400°C and the temperatures during combustion were below 600°C. The gauze temperature for the wood on its own reached 350°C and simply cooled without combustion SI4 Figure 9h).

SI4 Figure 9 : Combustion Process Temperatures

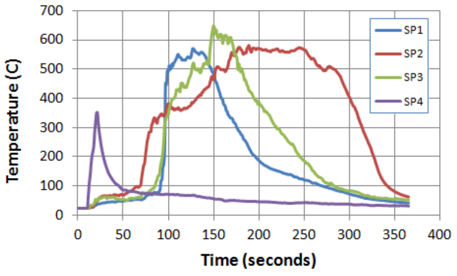

(a) MD6 - run code 066

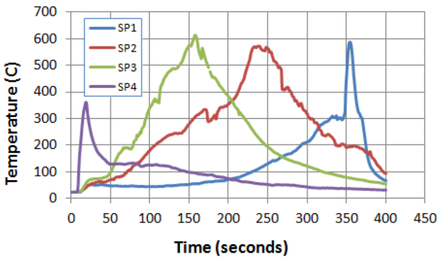

(b) MD5 - run code 064

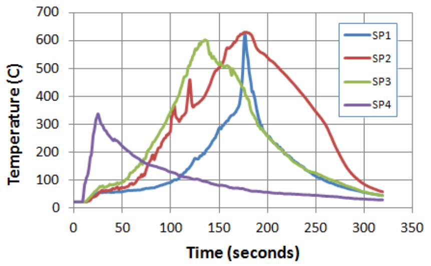

(c) MD4 - run code 063

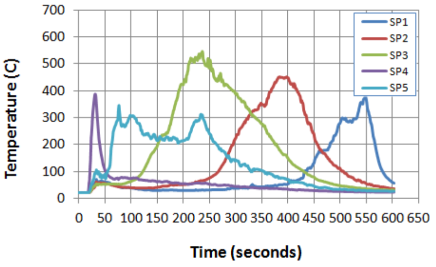

(d) MD3 - run code 090

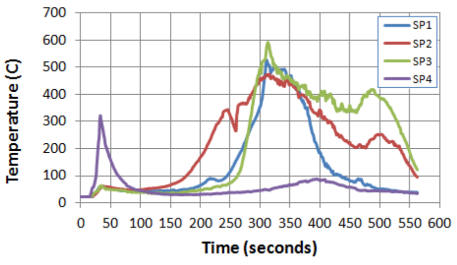

(e) MD2 - run code 089

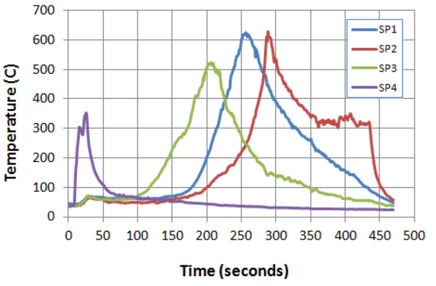

(f) MD1- run code 088

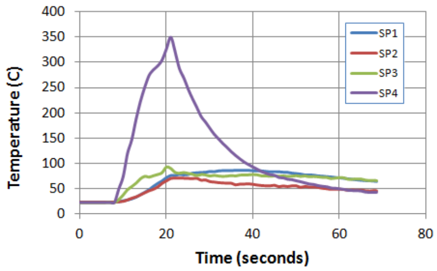

(g) Beech alone - run code 070

Supplementary Information 5 - TGA and DTG of Wood or Oxide and Wood Mixtures

DTG for Wood in Air and Nitrogen

Comparison of the DTG data for the beech wood in air and nitrogen (SI5 Figure 1) suggests there is some oxidative increase in the volatilization rate and probably combustion of some of the volatiles in air; there is a small drop in the peak pyrolysis temperature. The char produced by decomposition of the hemi-cellulose, cellulose and lignin undergoes combustion in air with a peak rate of combustion at around 460°C. Where char combustion is prevented by nitrogen, the residual mass of char was approximately 23% by weight.

SI 5 Figure 1 : DTG of Wood in Air and in Nitrogen (N_2)

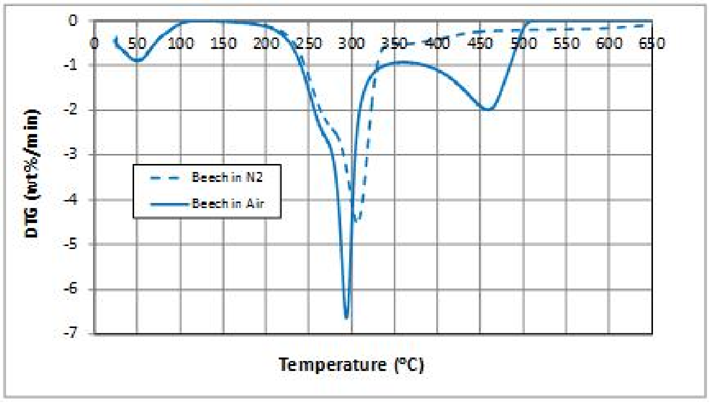

TGA and DTG for Romanèchite MD7 and Wood Mixture:

The DTG for the beech wood and MD7 romanèchite (SI5 Figure 2) indicates a reduced rate of pyrolysis and volatilization but a reduction in the temperature for the peak rate of char combustion to approximately 400°C.

The TGA is somewhat inconsistent with a much higher than expected residual mass at 650°C. The reasons for this behaviour are unclear but it might be that the MD7 has absorbed volatiles from the decomposing hemi-cellulose or cellulose.

SI 5 Figure 2 : DTG and TGA for MD7 and Wood Mixtures in Air

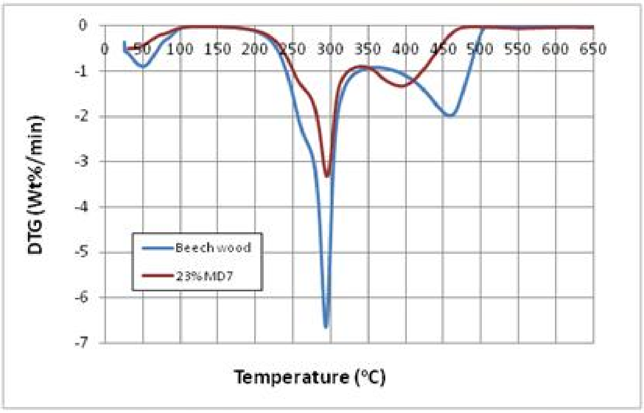

a. DTG

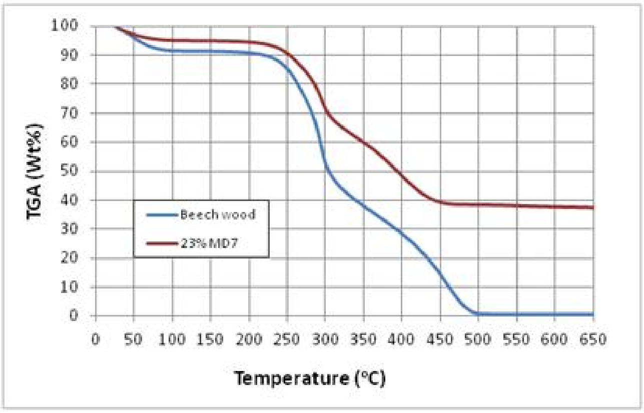

b. TGA

TGA and DTG for Manganese Dioxides and Romanèchite (without wood)

The TGA and DTG of the β-MnO_2 manganese dioxides MD3 and MD4 in SI5 Figure 3 are very different from the romanèchite MD7. Pech-de-l'Azé I *bloc* MD3 has the most sharply defined weight loss transition from MnO_2 to Mn_2O_3 in air at approximately 640°C. It also has a small transformation peak at approximately 220°C. The manganese dioxide MD4 decomposes over a somewhat wider range of temperatures centred at 555°C. MD7 loses substantially less mass and has less pronounced transitions. Unless romanèchite were to have an unexpectedly active interaction with reducing gases or volatiles, it would not produce the quantities of oxygen generated by the decomposition of manganese dioxide or influence auto-ignition of wood.

SI 5 Figure 3 : TGA and DTG for MD3, MD4 and MD7 in Air

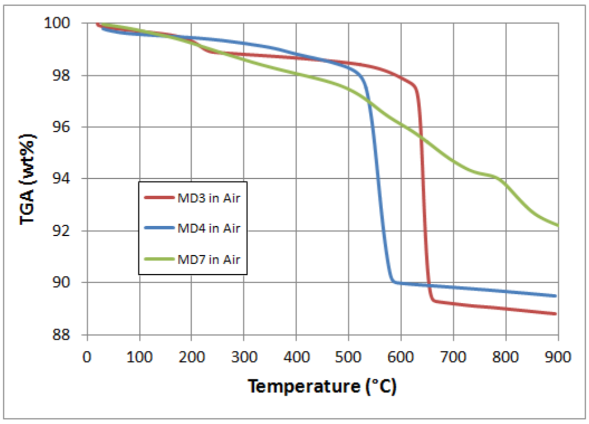

a. TGA

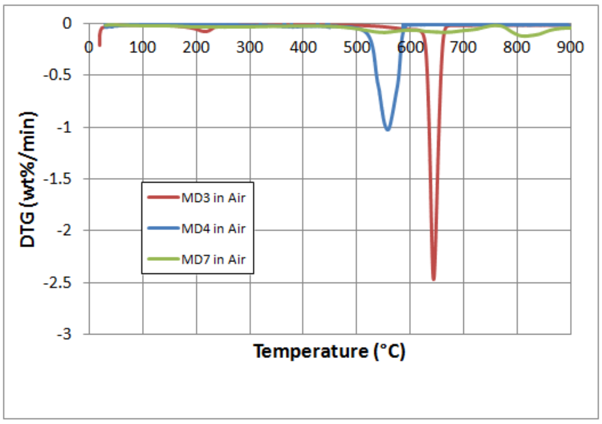

b. DTG

DTG for Lower Purity Manganese Dioxide (MD5) Mixtures with Wood

The DTG of the MD5 (approximately 82% manganese dioxide by XRF analysis) at 23% by weight with beech wood is similar to the higher MnO_2-content MD4 but with a lower rate of char combustion at 300°C (SI5 Figure 4). The rapid increase in the rate of combustion occurs at the temperature of the peak rate of pyrolysis, a slightly higher temperature than with MD4. At lower manganese dioxide to wood ratios, MD5 produced only a shift to lower char combustion temperatures. It may either be that the presence of large amounts of silicon, titanium and aluminium has an inhibiting effect on the interaction or that the lower concentration of manganese dioxide is important.

SI5 Figure 4 : DTG for MD5 and Wood Mixtures in Air

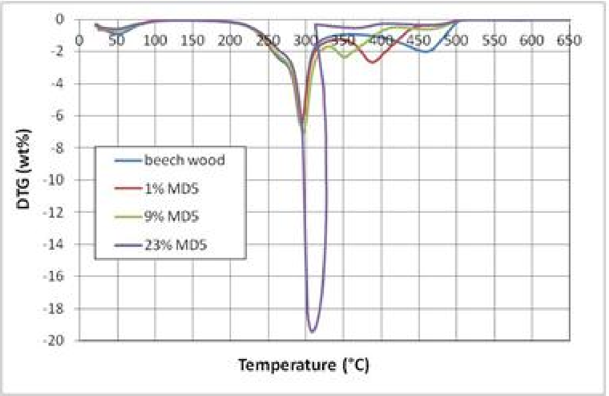

DTG for Manganese Dioxide MD6 and Wood Mixtures

The DTG for MD6 with beech wood (SI5 Figure 5) used in the thermal imaging fire experiments in Supplementary Information 4 is very similar to MD4. Both exhibit a very rapid weight loss at approximately 280°C before the normal peak in the rate of wood pyrolysis due to combustion of the wood char. Both are relatively pure manganese dioxide but MD4 has the β-MnO_2 (pyrolusite) structure whereas MD6 is amorphous. The thermal interaction with wood does not depend on the crystal structure.

SI5 Figure 5 : DTG for MD6 and Wood Mixtures in Air

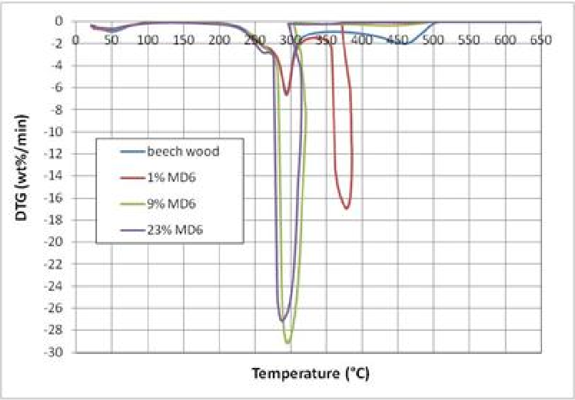

DTG for Pech-de-l'Aze I *Blocs* MD1 and MD2 in Mixtures with Wood

The DTGs for Pech-de-l'Azé I *blocs* MD1 and MD2 (SI5 Figure 6) show some of the variation in thermal response in the materials. MD1 reduces the char combustion temperature from 460°C to around 360°C without increasing the rate of combustion. MD2 reduces char combustion temperature to 360°C and increases the rate of char combustion seven-fold. In the fire experiments MD2 behaved much like MD3 and only MD1 required a longer heating time to ignite, but under the same conditions, the wood alone would not ignite.

SI5 Figure 6 : DTG for MD1 and MD2 Wood Mixtures in Air

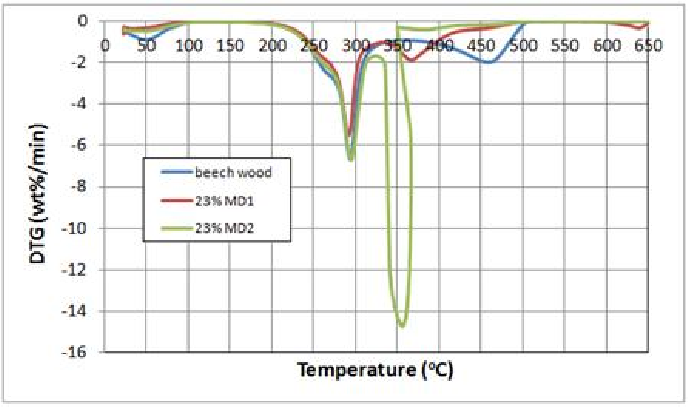

Supplementary Information 6 - Proposed Combustion Mechanism

We propose that the mechanism in facilitating combustion involves the low temperature decomposition of manganese dioxide, stimulated by the reactive gases derived from wood pyrolysis and the consequent release of oxygen that both reduces the critical temperature for ignition and increases the rate of char combustion.

Pure manganese dioxide with a β-MnO_2 structure decomposes on heating, releasing oxygen in several stages, to Mn_2O_3 at 631^oC, to Mn_3O_4 (hausmannite) at 963^oC and finally to MnO at temperatures above 1300^oC[21, 22]. The TGAs of the manganese dioxides without wood (Supplementary Information 5) show a first decomposition temperature of 555^oC for MD4 (96% MnO_2) and approximately 640^oC for the Pech-de-l'Azé I material MD3 and no further transition below 900^oC. However, in contrast to the very high temperatures required to produce hausmannite, XRD confirmed that the manganese dioxides and Pech-de-l'Azé I *blocs* transformed to hausmannite during combustion at the comparatively low temperatures in the combustion experiments (Figure 2 and Supplementary Information 4).

The presence of reactive gases has a strong effect on manganese dioxide decomposition, lowering the temperature of transformation to MnO from over 1300^oC to around 450^oC[23, 24, 25]. The pyrolysis of biomass materials has a similar effect, allowing manganese dioxide to decompose to MnO at temperatures between 300^oC and 500^oC[26]. In a similar manner, wood produces reactive, chemically-reducing volatile compounds as it undergoes thermal decomposition of the hemi-cellulose to volatile organic compounds followed at higher temperatures by the decomposition of the cellulose and lignin[27]. The beech wood used in the combustion experiments releases volatile organic compounds above 220^oC with a peak rate at 300^oC (Figure 4a). We suggest that the reducing gases and volatile organic compounds released in the initial decomposition of wood provide a reactive environment at the manganese dioxide surface that lowers the decomposition temperature to approximately 290^oC to 300^oC. The decomposition of manganese dioxide to hausmannite releases oxygen that reacts with the char, producing the glowing combustion. The overall effects of the manganese dioxide are a substantial reduction in the auto-ignition temperature of the wood and a substantially increased rate of combustion (Figure 4). Continuing combustion is supported by oxygen from the air. The initial thermal input for initiating wood pyrolysis, manganese dioxide decomposition and the modest temperatures required for auto-ignition can be produced with spark-lit tinder; under identical conditions wood alone does not ignite. The manganite (ϒ-MnOOH) found in most of the *blocs* transforms to manganese dioxide at 300^oC[18] and probably contributes to the combustion process.

Support for the mechanism is provided by the lack of effect shown by the thermally stable oxides, zinc oxide, aluminium oxide and titanium dioxide that do not release oxygen when heated to temperatures normally experienced in a wood fire.

Whereas manganese dioxide facilitates wood ignition, no such effect was found with wood and romanèchite (hydrated barium manganese oxide) mixtures. The lack of effect of romanèchite on wood ignition is perhaps explained by the significantly higher temperatures required for the decomposition of romanèchite, the lower amounts of oxygen released[22] and a reduced effect of wood decomposition volatiles on the decomposition process for romanèchite (see the DTGs for MD7 and MD7 and wood mixtures in Supplementary Information 5).

Supplementary References

1. Bordes, F.H. Sur l'usage probable de la peinture corporelle dans certaines tribus moustériennes. *Bulletin de la Société préhistorique de France*, 169-171 (1952).
2. Demars, P.Y. Les colorants dans le Moustérien du Périgord. L'apport des fouilles de F. Bordes. *Préhistoire Ariegeoise* **47,** 85-194 (1992).
3. Soressi, M. *et al*. in *Les sociétés du Paléolithique dans un grand Sud-Ouest de la France : nouveaux gisements, nouveaux résultats, nouvelles méthodes* (eds. Jaubert, J., Bordes, J.-G. & Ortega, I.) 95-132 (Société Préhistorique Française, 2008).
4. Bordes, F.H. *A Tale of two caves*.(Harper and Row Publishers 1972).
5. Laville, H., Rigaud, J. P. & Sackett, J. *Rock shelters of the Perigord: geological stratigraphy and archaeological succession*. (Academic Press, 1980).
6. Bordes, F. H. Le gisement du Pech de l'Azé IV. Note préliminaire. *Bulletin de la Société préhistorique française. Études et travaux* 293-308 (1975).
7. Dibble, H. L, *et al*. A preliminary report on Pech de l'Azé IV, layer 8 (Middle Palaeolithic, France). *PaleoAnthropology*, **2009,** 182-219 (2009).
8. Goldberg, P. *et al.* New evidence on Neandertal use of fire: examples from Roc de Marsal and Pech de l'Azé IV. *Quaternary International* **247,** 325-340 (2012).
9. Martin, H., 1923. *Recherches sur l'évolution du Moustérien dans le gisement de la Quina (Charente). Vol. 2 L'industrie lithique*. (Ouvrière, 1923).
10. Jelinek, A.J., *Neandertal lithic industries at La Quina*. (University of Arizona Press, 2013).
11. Peyrony, D. Le Moustier : ses gisements, ses industries, ses couches géologiques. *Revue Anthropologique* **40,** 3-76 and 155-176(1930).
12. San-Juan, C. Les matières colorantes dans les collections du Musée National de Préhistoire des Eyzies. *Paléo* **2,** 229-242 (1990).
13. Valladas, H., *et al*. Datation par la thermoluminescence de gisements moustériens du sud de la France. *L'Anthropologie* **91,** 211-226n (1987).
14. Pradel, L. J.H. Le Mousterien évolué de l'Ermitage. *L'Anthroplogie* **58,** 433-443 (1954).
15. Bordes, F.H. La stratigraphie de la Grotte de Combe-Grenal, commune de Domme (Dordogne) Note préliminaire. *Bulletin de la Société Préhistorique de France*, 426-429 (1955).
16. Faivre, J. P. *Organisation techno-économique des systèmes de production dans le Paléolithique moyen récent du Nord-est Aquitain: Combe-Grenal et les Fieux*. Bordeaux (Université Bordeaux 1 Thesis, 2008).
17. Anthony, J.W., Bideaux, R.A., Bladh, K.W. & Nichols, M.C. (eds). *Handbook of Mineralogy*. (Mineralogical Society of America, 2014).
18. Kohler, T., Armbruster, T. & Libowitzky, E. Hydrogen bonding and Jahn-Teller distortion in groutite, α-MnOOH, and manganite, γ-MnOOH, and their relations to the manganese dioxides ramsdellite and pyrolusite. *J. Solid State Chem.* **133,** 486-500 (1997).
19. Heyes, P.J. *Pragmatic or symbolic: Neanderthals uses for manganese dioxide in South West France during the late Middle Palaeolithic*. (Leiden University Thesis, 2014).
20. Chalmin, E. *Caraterisation des Oxydes de Manganese et Usage des Pigments Noirs au Paleolithique Superieur*. (Université de Marne-La-Vallée Thesis, 2003). https://tel.archives-ouvertes.fr/tel-00120355/document; accessed Mar 2014.

21. Li, L., Hea, M., Zhang, A. & Zhou, J. A study on non-isothermal kinetics of the thermal decompositions of β-manganese dioxide. *Thermochim. Acta* **523,** 207– 213 (2011).
22. Bish, D.L. & Post, J.E. Thermal behaviour of complex, tunnel-structure manganese oxides. *Am. Min.* **74,** 177-186 (1989).
23. Zaki, M.I., Hasan, M.A., Pasupulety, L. & Kumari, K. Thermochemistry of manganese oxides in reactive gas atmospheres: Probing redox compositions in the decomposition course MnO2 to MnO. *Thermochim. Acta* **303,** 171-181 (1997).
24. Zaki, M.I., Hasan, M.A., Pasupulety, L. & Kumari, K. Thermochemistry of manganese oxides in reactive gas atmospheres: Probing catalytic MnOx compositions in the atmosphere of CO plus O2. *Thermochim. Acta* **311,** 97-103 (1998).
25. Gonzalez, C. *et al*. Transformations of manganese oxides under different thermal conditions. *J. Therm. Anal.* **47,** 93-102 (1996).
26. Zhang, H., Zhu, G., Yan, H., Li, T. & Feng, X. Thermogravimetric analysis and kinetics on reducing low-grade manganese dioxide ore by biomass. *Metall. Mater.Trans. B* **44B,** 878-888 (2013).
27. Muller-Hagedorn, M., Bockhorn, H., Krebs, L. & Muller, U. A comparative kinetic study on the pyrolysis of three different wood species. *J. Anal. Appl. Pyrol.* **68/69,** 231-249 (2003).
